# On the Search of a Silver Bullet for the Preparation of Bioinspired Molecular Electrets with Propensity to Transfer Holes at High Potentials

**DOI:** 10.3390/biom11030429

**Published:** 2021-03-15

**Authors:** James Bennett Derr, Katarzyna Rybicka-Jasińska, Eli Misael Espinoza, Maryann Morales, Mimi Karen Billones, John Anthony Clark, Valentine Ivanov Vullev

**Affiliations:** 1 Department of Biochemistry, University of California, Riverside, CA 92521, USA; jderr002@ucr.edu; 2 Department of Bioengineering, University of California, Riverside, CA 92521, USA; katarzyna.rybickajasinska@gmail.com (K.R.-J.); jclar019@ucr.edu (J.A.C.); 3 Department of Chemistry, University of California, Riverside, CA 92521, USA; eespi009@berkeley.edu (E.M.E.); mmorale2@caltech.edu (M.M.); 4 Department of Biology, University of California, Riverside, CA 92521, USA; mbill010@ucr.edu; 5 Department of Materials Science and Engineering Program, University of California, Riverside, CA 92521, USA

**Keywords:** Purdie-Irvine alkylation, etherification, molecular electrets, dipoles

## Abstract

Biological structure-function relationships offer incomparable paradigms for charge-transfer (CT) science and its implementation in solar-energy engineering, organic electronics, and photonics. Electrets are systems with co-directionally oriented electric dopes with immense importance for CT science, and bioinspired molecular electrets are polyamides of anthranilic-acid derivatives with designs originating from natural biomolecular motifs. This publication focuses on the synthesis of molecular electrets with ether substituents. As important as ether electret residues are for transferring holes under relatively high potentials, the synthesis of their precursors presents formidable challenges. Each residue in the molecular electrets is introduced as its 2-nitrobenzoic acid (NBA) derivative. Hence, robust and scalable synthesis of ether derivatives of NBA is essential for making such hole-transfer molecular electrets. Purdie-Irvine alkylation, using silver oxide, produces with 90% yield the esters of the NBA building block for iso-butyl ether electrets. It warrants additional ester hydrolysis for obtaining the desired NBA precursor. Conversely, Williamson etherification selectively produces the same free-acid ether derivative in one-pot reaction, but a 40% yield. The high yields of Purdie-Irvine alkylation and the selectivity of the Williamson etherification provide important guidelines for synthesizing building blocks for bioinspired molecular electrets and a wide range of other complex ether conjugates.

## 1. Introduction

In its many forms, charge transfer (CT) is the fundamental process that sustains life on Earth and makes our modern ways of living possible [1]. Biological, biomimetic, and bioinspired systems encompass some of the most important templates for advancing CT science and engineering [2]. The Marcus semiclassical transition-state theory has revolutionized the understanding of biological CT and bioenergetics at a molecular level [3]. The diabatic nature of CT mediated by biomolecules has made Marcus-Hush and Marcus-Levich-Jortner formalisms perfect for the analysis of such systems [1]. At the same time, biological CT has driven the evolution of CT science, resulting in new theories and unprecedented ways of thinking [4,5,6,7,8,9].

**Figure 1:**
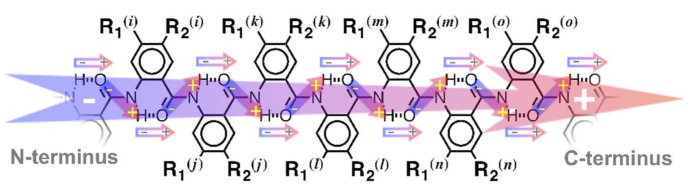
Bioinspired molecular electrets composed of anthranilamide (Aa) residues and the origin of their macrodipoles from ordered arrangement of amides and polarization upon the formation of hydrogen bonds.

The ubiquity of electric dipoles makes the understanding how they affect CT crucially important not only for biology, but also for materials science and device engineering [10]. The first ideas about dipole effects on CT originated in the mid-20th century from considerations of CT through protein structures with numerous polar moieties [11,12]. In the 1990s, CT through biological and bioinspired molecules provided the experimental evidence for such effects [13,14,15,16]. As the field took off, the focus was on dipolar biomolecules on one hand, and on small polar organic conjugates on the other, limiting the range of CT to 2 nm, and the mechanism predominantly to tunneling or superexchange [1,6,10]. To take the field out of this confinement, we developed the concept for CT bioinspired molecular electrets (Figure 1) [17,18,19,20,21,22,23,24,25,26,27,28,29].

Comprising ordered electric dipole moments, electrets are the electrostatic analogues of magnets. Some of the best molecular electrets are biomolecules such as protein α-, 3_10_-, and polyproline I helices [30]. Peptide bonds (i.e., aliphatic N-acyl amides) are a small group with substantial permanent dipole moments [31]. Therefore, the arrangement of peptide bonds in protein helical folds results in macrodipoles of about 4 D per residue [30]. Hydrogen bonding in α-, and 3_10_-helices shifts the electron density from the carbonyl oxygens on one loop to the amide protons on the next one. This polarization further enhances the macrodipoles, which for protein α-helices can exceed 5 D per residue with an increment of 0.15 nm. That is, 3-nm stretches of α-helices, comprising 20 amino acids arranged in about 5.6 loops, have dipoles of 100 D, which generate electric fields of tenths of GV m^−1^ in the vicinity of these biomolecules. It does not come as a surprise, therefore, that polypeptide helices have been the principal templates for investigating dipole effects on CT [32,33,34,35,36].

The propensity for oxidative degradation of amides, however, limits CT to about a 2-nm range attainable by electron tunneling through such biomolecules [37,38,39]. Potentials of about 1.4–1.5 V vs. saturated calomel electrode (SCE), indeed, allow for hole injection in peptide bonds. A couple of σ-bonds separate each two peptide bonds in proteins, decreasing the electronic coupling between them by a factor of eight. It results in hole residence times on the amides that are long enough to permit their oxidative degradation, as evident from the irreversibility of their electrochemical oxidation [40,41].

When it comes to efficient long-range hole transfer through insulating materials, another class of biomolecules present some of the best paradigms. Deoxyribonucleic-acid (DNA) and peptide-nucleic-acid (PNA) efficiently transfer holes over tens of nanometers along their π-stacked electron-rich bases [42].

Considering the desirable features of these two completely different classes of biomolecules (i.e., protein helices and DNA/PNA strands), we designed bioinspired molecular electrets based on anthranilamide (Aa) structural motifs (Figure 1) [28].

Like for protein helices, the ordered amide and hydrogen bonds of the bioinspired electrets generate a macrodipole of about 3 D per residue [17,19]. The Aa aromatic moieties not only provide hopping sites for electrons or holes to ensure long range CT, but also take away a part of the electron density from the amide bonds to prevent oxidative degradation [22]. The side chains, R_1_ and R_2_ (Figure 1), of the non-native Aa amino acids play a crucial role in controlling their electronic properties. Electron-donating R_2_ substituents stabilize the radical cations of such Aa residues by pulling the spin density away from their C-terminal amides, making them excellent candidates for mediating hole transfer via long-range hopping. Electron-donating substituents, however, also lower the reduction potentials of these radical cations, precluding the transduction of holes with high enough energy to drive certain chemical transformations or to ensure large voltages at the termini of energy-conversion devices [1,43,44]. Therefore, we focused on ether-functionalized Aa conjugates. Ethers as R_2_ substituents are electron-withdrawing enough to ensure stability of the Aa radical cations, but not too polarizing to lower their reduction potential to ≤1 V vs. SCE, as we observe for amines [20,24]. As we show, aromatic ether substituents such as R_2_, sterically locked in orthogonality with the Aa ring, lead to reversible electrochemical oxidation at about 1.7 V vs. SCE at scan rates of 100 mV s^−1^ or less [27]. Upon increasing the scan rates beyond 0.5 V s^−1^, aliphatic and other aromatic ethers of Aa residues commence to show reversibility at a potential between 1.5 and 1.9 V vs. SCE [27]. Considering the timescales of electrochemical measurements ensures that the oxidative degradation of the ether Aa residues showing even partial reversibility is orders of magnitude slower than the residence time of holes on them during CT through Aa electrets. Furthermore, the potentials exceeding 1.5 V vs. SCE are the highest known under which amides can undergo reversible, or even partially reversible, oxidation [27].

**Figure 2:**
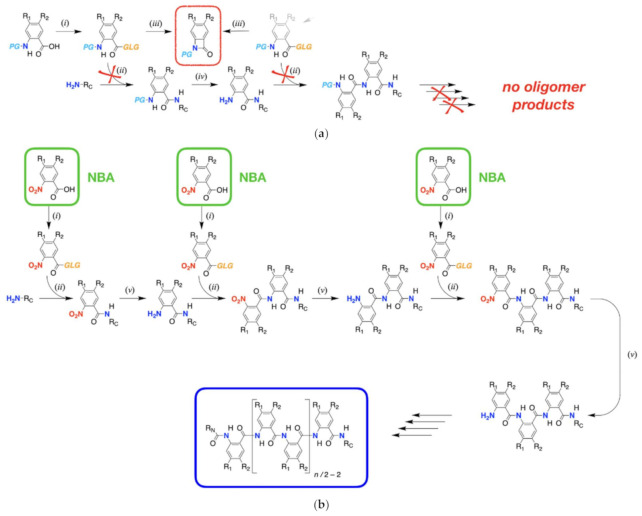
Synthesis of bioinspired molecular electrets composed of Aa residues. (**a**) An established protocol for peptide synthesis where each residue is introduced as its protected-amine derivative, with a protection group (*PG*) such as Fmoc or *t*Boc. Ideally, the synthesis of the polyamide molecular electrets should involve a sequence of repetitions of carboxylate-activation (*i*) and amide-coupling (*ii*) steps, followed by amine deprotection (*iv*). The enormous efficiency of the intramolecular formation of stable cyclic *β*-lactams (in the red frame) (*iii*), however, precludes the intermolecular amide coupling (*ii*), rendering this synthetic strategy unacceptable. (**b**) An alternative protocol for the synthesis of Aa molecular electrets with *n* Aa units (in the blue frame), capped with R_N_ and R_C_ at the N- and C-termini, respectively, where each residue is introduced as its 2-nitrobenzoic acid (NBA) derivative (in the green frames). The multistep synthesis involves repetitions of carboxylate activation (*i*), amide coupling (*ii*), and nitro reduction (*v*). Steps (*i*) and (*ii*) can be carried out separately or in the same pot involving in situ carboxylate activation in the presence of the free amine. Specifically, the involved reaction steps are: (*i*) activation of the carboxylic acid by attaching a good living group (*GLG*) to the electrophilic carbonyl carbon via: (*i.*1) halogenation (i.e., *GLG* = -Cl or -F); (*i.*2) a reaction with a carbodiimide reagent in the presence of hydroxyl derivatives such as *N*-hydroxysuccinimide (HOSu) (i.e., *GLG* = -OSu, 1-hydroxybenzotriazole (HOBt), i.e., *GLG* = -OBt, or 1-hydroxy-7-azabenzotriazole (HOAt), i.e., *GLG* = -OAt); (*i.*3) a reaction with an onium reagent such as HOBT or HATU in the presence of HOBt or HOAt; (*ii*) coupling between the activated acid and a primary (or secondary) amine (i.e., amide coupling); (*iii*) formation of a stable cyclic β-lactam (in the red frame): the close proximity of a weakly nucleophilic nitrogen (in a protected amine or in an amide) at the ortho position to the activated carboxylate makes it immensely prone to intramolecular amide coupling leading to a cyclic lactam; (*iv*) deprotection of the amine (e.g., using base for Fmoc *PG*, and acid for *t*Boc *PG*), and (*v*) selective reduction of the N-terminal nitro group to an amine.

A principal task in this line of research was making the Aa bioinspired molecules [28,29], which is the focus of this publication. While they are polypeptides of aromatic β-amino acids, none of the strategies for chemical or biochemical synthesis of biological and biomimetic peptides works for making Aa conjugates (Figure 2a) [29]. Using Fmoc- or *t*Boc-protected derivatives, or any form of amine or amide at the ortho positions next to the activated carboxylate has two detrimental effects: (1) lowers the electrophilicity of the carbonyl carbon of the activated acid, which lessens the efficiency of the amide coupling to the N-terminal amine; and (2) efficiently drives the formation of cyclic β-lactams that are pronouncedly stabile (Figure 2a). As we show, only strong nucleophiles such as piperidine can open these cyclic lactams [29], rendering them impractical for the synthesis of Aa conjugates.

To build the Aa strands from the C- to the N-terminus, we introduced each of the amino acids as its 2-nitrobenzoic acid (NBA) analogues (Figure 2b). Instead of introducing an *N*-protected amino acid at each step, which is selectively deprotected for attaching the next one, we coupled a NBA analogue to the N-terminal amine and selectively reduced -NO_2_ to -NH_2_ to prepare it for the attachment of the next residue (Figure 2b) [29]. Addition of the residue building blocks such as NBA derivatives via a sequence of amide-coupling and nitro-reduction steps yields Aa oligomers of different lengths and complexity [29]. Alkyloxy derivatives of NBA are essential for building ether-functionalized Aa conjugates to serve as molecular electrets that can mediate the transfer of high-energy holes via hopping (i.e., incoherent CT that has the key advantage of covering long distances) [1]. The capability to make highly pure NBA derivatives in large quantities, with as few synthetic and purification steps as possible, is essential for advancing the pursuit for new Aa bioinspired molecular electrets.

Nucleophilic aromatic substitution of fluorinated NBAs in a solvent-free environment allows for amine derivatives of NBA to be obtained in excellent yields [18,20,24]. Employing aliphatic amines with long alkyl chains, however, places a certain demand on this synthetic procedure such as the use of microwave heating to drive the reaction. Unlike conventional heating, microwave radiation aids in overcoming the entropic contributions to the transition-state energy where only a few of the enormous possible conformers of the collision complexes between the starting materials lead to the desired product.

Employing nucleophilic aromatic substitution for the synthesis of alkyloxy NBAs, however, has proven utterly unfeasible. Fluorinated NBA derivatives can react with phenoxy nucleophiles in the presence of a carbonate base in organic solvent to produce aromatic ethers of NBA [27]. Unfortunately, the same NBAs decompose in the presence of alkyloxy nucleophiles, requiring strong bases or alkaline metals to generate them. Therefore, we resorted to etherification of hydroxyl derivatives of NBA with alkyl halides. This leads to the ether-ester derivatives of NBA, which sets the requirement for an additional step in the synthesis: hydrolysis to convert the ether-ester derivative to the corresponding free acid ether-NBAs needed for the synthesis of the electrets (Figure 2b) [20].

Recently, we discovered a variation of Williamson etherification (WE) that selectively yields ether derivatives of NBA without ester products [27]. The most crucial requirement of our WE is the solvent, 2-methoxyethanol (2ME), which selectively produces ether NBAs in good to excellent yields [27]. Other alcoholic solvents produce only traces of the desired products, at best. Employing neat conditions (where the alkyl halide acts as a reagent and solvent) does not lead to even traces of the products [27]. These outcomes warrant the search for alternative etherification procedures under mild conditions that do not necessarily involve strong bases.

As a variation of WE, Purdie-Irvine alkylation (PIA), also referred to as Purdie methylation and Irvine-Purdie methylation, accommodates the reaction of alcohols with alkyl halides in the presence of solid silver oxide, Ag_2_O [45]. In spite of the disputes over the exact name of this reaction, A. Wurtz was the first to report such Ag_2_O-driven ether formation from different iodides and alcohols about half a century before Purdie and Irvine published their findings [46]. Decades of work on the esterification of silver and mercury salts of carboxylic acids with alkyl iodides provided an important platform for Purdie’s research, establishing Ag^+^ and aliphatic halides as a promising combination of reagents for alkylation [47].

Despite some misconceptions [48], Ag_2_O is not a catalyst, but a consumable reactant where the strong affinity of Ag^+^ for halide ions drives the reaction forward (i.e., 2R′X + 2ROH + Ag_2_O → 2R′OR + 2AgX + H_2_O). Nevertheless, PIA can still benefit from adding catalysts [49]. Silver oxide is a weak Lewis base that, while aiding the process by neutralizing the released protons, makes PIA an excellent choice for etherification involving base-sensitive reactants and products. Furthermore, prolonged reaction times allow PIA to achieve quantitative yields under relatively mild conditions. These desirable features have established PIA as the preferred method for the methylation of sugars [50,51,52,53], which is essential for carbohydrate analysis and have proven important for the development of glycomics [54,55,56,57]. With few exceptions [58,59,60], however, PIA remains largely unexplored beyond the methylation of carbohydrates, which Purdie and Irvine described more than a century ago [45,53].

**Figure 3:**
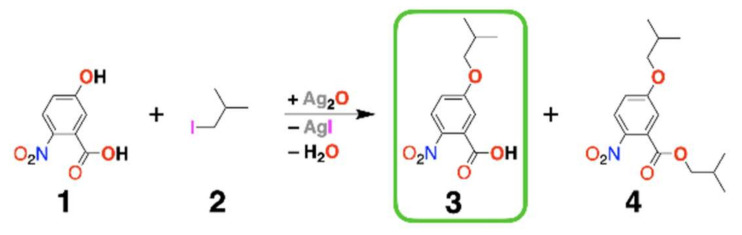
Purdie-Irvine alkylation (PIA) leading to an ether 2-nitrobenzoic acid (NBA) precursor (in the green frame) for the synthesis of Aa bioinspired molecular electrets (Figure 1; Figure 2b). The exact masses of the products, obtained using as high-resolution mass spectrometry (HRMS), along with their ^1^H and ^13^C nuclear magnetic resonance (NMR) spectra (Figure S1 and S2), confirm the identities of **3** and **4**.

Herein, we present an investigation of the utility of PIA for the synthesis of ether NBA derivatives under neat conditions where iso*-*butyliodide acts as a reagent and a solvent (Figure 3). Microwave heating leads to the formation of the ether derivative of NBA, **3**, and its ester, **4**, with overall yield of 40%. PIA employing conventional heating, on the other hand, produces ester **4** in 90% yield. With ether **3** in hand, we demonstrate the synthesis of electret oligomers that revealed substantial differences in the reactivities for coupling different residues together. These findings about PIA and on building the oligomers provide key guidelines for the design of the synthesis not only of bioinspired molecular electrets, but also for a wide range of ether derivatives.

## 2. Results and Discussion

### 2.1. Choice of Substrates

The synthesis of Aa molecular electrets involves adding each residue as its NBA analogue from the C- to the N-terminus [29]. The ortho nitro group in the NBA derivatives enhances the electrophilicity of the carbonyl carbon of the activated carboxylate. It is essential for coupling the NBA with the N-terminal amine (i.e., the amine of the previous residue), which is not strongly nucleophilic and experiences some steric hindrance. Activating the NBAs as acyl chlorides provides the necessary reactivity. Furthermore, the small size of -COCl is beneficial for attacking the N-terminal aromatic amine next to an *ortho* positioned amide. Therefore, synthesis of NBA analogues of the Aa residues is the first and most important step in the preparation of the bioinspired molecular electrets.

Placing ethers at the fifth position of Aa residues (R_2_, Figure 1) allows for attaining reversible oxidation at unusually high potentials for amides [27]. Our recent electrochemical studies showed that placing a 2,6-dimethylphenyloxy substituent at position 5 provided the most pronounced stability of the Aa radical cation at potentials that no amide has been previously known to sustain [27]. Alkyloxy-functionalized Aa residues manifest only partial reversibility during electrochemical oxidation. Nevertheless, the aliphatic sidechains of such Aa ethers substantially improve their solubility in organic media, which is a key consideration for the design of Aa oligomers to facilitate the work with them. Etherification of 5-hydroxy-2-nitrobenzoic acid (**1**, Figure 3) with alkyl halides provides the best route for synthesizing the NBA precursors for such Aa derivatives.

Attaching an ether with a long aliphatic flexible chain substantially improves the solubility of such derivatives. Leftover alkyl halide starting martials with a large molecular weight are not volatile enough to be readily removed from the reaction mixture, warranting the employment of chromatography [20]. While light-weight alkyl halides can be readily removed in vacuo at low temperature, their short aliphatic chains would not prove too beneficial for solubilizing the Aa oligomers. Butyl halides present the best compromise because they are small enough to readily remove them from the reaction mixtures. At the same time, butyls are large enough chains, especially when branched, to ensure sufficient solubilization of the ether Aa conjugates in organic solvents. The iso*-*butyl side chain of leucine **(Leu)** ensures substantial lipophilicity Leu-rich protein segments [61,62,63,64,65,66,67,68,69,70,71,72,73,74,75,76]. These side chains also drive the formation of leucine zippers as a part of amphipathic motifs that hold tertiary and quaternary protein structures together. Therefore, etherification of **1** with *iso-*butyliodide (**2**, Figure 3) presents an excellent choice for examining the utility of PIA for making ether Aa derivatives.

### 2.2. Synthesis Design

PIA is immensely important for methylation of sugars in aqueous and other polar media. Using methyl iodide leads to the formation of methyl ethers where hydroxyls previously were. Employing it in polysaccharides, followed by their hydrolysis, to the comprising monomers provides access to useful structural information [77]. The polarity and the hydrogen-bonding propensity of the aqueous media is essential for dissolving the carbohydrates and for lowering the transition-state energy essential for attaining acceptable reaction rates. Such conditions, however, are not feasible for the etherification of alcohols and phenols that are not soluble in water. It warrants the use of an organic solvent, providing a good compromise between dissolving the hydroxyl reactants and suspending the solid Ag_2_O.

Conducting the PIA reaction under neat conditions provides an attractive choice for pursuing green-chemistry strategies where scaling up does not require an increase in solvent consumption. The capability to suspend the NBA reactant, **1**, and the solid Ag_2_O in the alkyl halide, **2**, while keeping the reaction mixture fluent enough to stir, sets the limits on the minimum amounts of halide needed. Our aim at butylation, rather than methylation, of **1** requires the suspension of the reaction mixture in **2**, which is a less polar solvent than methyl iodide. Nevertheless, using ×8 to ×9 molar excess of **2**, allows the other two reactants to be suspended in it for heating in a microwave reactor. Eight-to-nine fold excess of the liquid halide may seem excessive when claiming the benefits of solvent-free conditions. Nevertheless, it exceeded by only about a factor of two excess of **2** for etherification of **1** in an optimized solvent environment. When employing 2ME as a solvent, we used four times excess of the halide, **2**, for etherification of **1** in the presence of Cs_2_CO_3_ [27].

The optimal temperature for the microwave-driven PIA between **1** and **2** was 130 °C at 60 W exerted for two intervals of 30 s. This high temperature, exceeding the boiling point of **2** by about 10 °C, is necessary for the reaction to proceed, but must be halted after 30 s to prevent the risk of evaporating the halide too fast, which can cause splashing of the reaction mixture out of the vessel. Further microwave irradiation does not improve the reaction yields. The capabilities of microwave radiation to induce localized heating, especially for heterogeneous reactions with solids that have strong absorptivity for GHz electromagnetic waves, indicates that what we recorded using infrared sensors is an average temperature. That is, while the bulk of the halide **2** could be relatively cool because it is not a strong microwave absorber, at the Ag_2_O surface, the local temperatures can be exuberantly high.

These microwave synthetic conditions lead to the formation of the desired NBA ether, **3**, and its ester derivative, **4** (Figure 3, Scheme S1). Doubling the loading of Ag_2_O from 1 to 2 equivalents, relevant to **1**, increased the overall yield by about 50% (i.e., from 27% to 40%) while doubling the yield of **3** (i.e., from 9% to 20%) (Table 1). An increase in the oxide loading beyond two equivalents requires extra amounts of **2** to keep the suspension fluent, which decreases the concentration of **1** and does not truly improve the yields of the isolated product. Needless to say, increasing the Ag_2_O loading also challenges the cost-efficiency of the procedure. The two equivalents of oxide show the need of four Ag^+^ cations for each I^−^ anion produced. While it may appear excessive, it should be considered that most of the silver ions are inside the Ag_2_O solid and not exposed to the liquid phase of the reaction.

These findings, along with the results about WE from our previous work [27], reveal an important mechanistic insight. Reacting **1** and **2** under microwave radiation in the presence of Cs_2_CO_3_ suspended in 2ME also gave a 40% yield, but only of **3**, with no traces of **4** [27]. That is, Ag_2_O under neat conditions and the carbonate in 2ME drove the conversion of **1** to products with quite similar propensities. The alkaline carbonate was more basic than Ag_2_O, and 2ME was protic and more hygroscopic than 2. Hence, Cs_2_CO_3_ suspension in 2ME provided more favorable conditions for base-catalyzed irreversible ester hydrolysis than Ag_2_O suspension in **2**. This shows that the selectivity of the formation of **3** over **4**, which 2ME, and no other solvent, induces, is not due to the suppression of the carboxylate nucleophilicity or the halide reactivity. The p*K_a_* of 4-nitrophenol is about 7 and of 2-nitrobenzoic acid is about 2. Additionally, the nitro group dominates the electronic properties of **1**, considering the Hammett constants for the three substituents: *σ_p_*(NO_2_) = 0.78; *σ_m_*(CO_2_H) = 0.37; and *σ_m_*(OH) = 0.12 [78]. This indicates that under any conditions where the hydroxyl of **1** is deprotonated and can act as a good nucleophile, its carboxyl is also deprotonated, warranting the formation of ester under any conditions that favor etherification. What the 2ME/Cs_2_CO_3_ medium offers are conditions for fast irreversible hydrolysis of **4** to **3**, rather than the selectivity of forming **3** over **4** (i.e., the conditions for etherification of **1** always favor the production of ester derivatives). Once again, why is 2ME such a unique solvent for making ether derivatives of NBA from **1** [27]? Using media with stronger basicity than the carbonate suspension in 2ME will favor the hydrolysis, but (1) will interfere with the etherification by reacting with the halide, for example, and (2) lead to degradation of the formed products. Conversely, too low a basicity would fail to deprotonate the phenol, which is needed for making it a good nucleophile for etherification. In addition, considering the kinetic aspects of the processes suggests that at the reflux temperature of the 2ME/Cs_2_CO_3_ mixture, the etherification, esterification, and hydrolysis are faster than the undesired side reactions. Indeed, using other alcohols as solvents leads to traces of products, but nothing like the conversion yields observed for 2ME [27]. In its uniqueness, the **2**/Ag_2_O medium is almost as good as the 2ME/Cs_2_CO_3_ mixture. While the weak basicity of Ag_2_O does not drive the hydrolysis of **4** to **3** to completion, the strong affinity of Ag^+^ ions for halides ensures efficient alkylation of the hydroxyl and the carboxyl.

### 2.3. Testing Beyond Ag_2_O

Among all readily available compounds, Ag_2_O is the richest in silver cations (i.e., 93% of the weight of Ag_2_O is from Ag^+^). Nevertheless, the use of two equivalents of silver oxide added for each mole of **1**, and considering the product yields (20% of **3** and 20% of **4**) giving 0.8 equivalents of I^−^ based on the assumption that all of **3** originates from the hydrolysis of initially formed **4**, suggests that only 20% of the Ag^+^ ions are consumed in the formation of AgI (Figure 3). While this appears to be a small number, a lot less than 20% of the silver ions are on the surfaces of the micrometer size Ag_2_O particles used for this reaction. Therefore, the process involves silver ions not only on the surface, but also in the bulk of Ag_2_O. The need for accessing the bulk ions can be a considerable hurdle for the reaction.

The lack of solubility of Ag_2_O in organic media and the needed five-fold excess of silver ions suggests that most of the Ag^+^ cations, in the bulk of the crystal lattice, are not easily accessible to the other reagents and to the hydrogen iodide that forms. To examine the potential effects of solubilizing the inorganic reactant, we carried out the same procedure but instead of Ag_2_O, we used silver salts with different solubilities in organic media. Microwaving a mixture of **1** and **2** in the presence of some of the most readily available silver compounds, Ag_2_SO_4_, AgNO_3_, Ag(CH_3_CO_2_), and Ag(CF_3_SO_3_) produced neither **3** nor **4**. After the heating treatment, the reaction mixtures predominantly contained the starting materials **1** and **2**, along with small amounts of side products that were not **3**, **4**, or an ester of **1** with a free hydroxyl. While Ag(CH_3_CO_2_) and Ag(CF_3_SO_3_) are soluble in organic solvents, Ag_2_SO_4_ and AgNO_3_, are not. Hence, the solubility of the silver compound is not a deterministic factor for the PIA of **1**.

Reacting silver carboxylates with aliphatic halides is an established method for esterification. The weakly basic nature of Ag_2_O makes it prone to dissolution by acidic compounds such as **1** to form the corresponding silver salts (e.g., Ag^+^**1**^−^ and Ag^+^_2_**1**^2−^). Reactions of silver phenoxides and carboxylates with aliphatic halides should generate ethers and esters, respectively. If the formation of silver salts of **1** is necessary for PIA, Ag_2_SO_4_, AgNO_3_, and Ag(CF_3_SO_3_) should not drive the conversion to **3** and **4** because of the negative p*K_a_* values of sulfuric, nitric, and triflic acids. That is, nitrobenzoic acids and nitrophenols cannot protonate sulfate, nitrate, and triflate anions, rendering the formation of silver salts of **1** from Ag_2_SO_4_, AgNO_3_, and Ag(CF_3_SO_3_) impossible.

Following this line of thinking suggests that Ag(CH_3_CO_2_) should lead to at least some alkylation of **1** because the p*K_a_* of acetic acid is larger than the p*K_a_* of 2-nitrobenzoic acid. Nevertheless, Ag(CH_3_CO_2_) is as inefficient for driving the conversion as the other silver salts. This finding points to the fact that the desirable solubility of Ag(CH_3_CO_2_) in organic media actually originates from the stable non-charged cyclic complex, disilver diacetate, in which Ag(CH_3_CO_2_) preferentially exists. Hence, breaking the coordination of the silver ions with the bidentate acetate ligands in [Ag_2_(CH_3_CO_2_)_2_] in order to form silver salts of **1**, requires from **1** to form complexes with Ag^+^, which have stability and solubility in non-polar media that are comparable to those of disilver diacetate. The requirement for solubility in **2** is especially challenging for species with doubly charged ions such as Ag^+2^**1**^2−^. In addition, the reactivity of the acetate ion under these alkylation conditions can make [Ag_2_(CH_3_CO_2_)_2_] + 2 IC_4_H_9_ → 2 CH_3_CO_2_C_4_H_9_ + 2 AgI the dominating reaction.

While esterification reactions involving silver carboxylate salts may provide an appealing premise for the need to dissolve Ag_2_O by **1**, the efficiency of PIA involving aliphatic alcohols renders this requirement as unnecessary. The weak acidity of alkyl alcohols precludes the possibility of dissolving the solid Ag_2_O in the form of salts (i.e., silver alkoxides). Therefore, structural considerations of the solid materials can prove important for understanding why Ag_2_O is uniquely efficient for driving this heterogeneous PIA. Later in this section, the discussion will focus on comparing the crystal-lattice characteristics of the starting material, Ag_2_O, and the produced AgI (Table 2).

Why are the silver ions so unique for this type of etherification? Along with Hg_2_^2+^, Pb^2+^, and Tl^+^, Ag^+^ is in the first analytical group of cations, characterized by the propensity for forming halides with pronouncedly small solubility products for aqueous media (e.g., *K_sp_*(Hg_2_I_2_) = 3.4 × 10^−28^ M^3^, *K_sp_*(PbI_2_) = 4.5 × 10^−9^ M^3^, *K_sp_*(AgI) = 2.0 × 10^−16^ M^2^, and *K_sp_*(TlI) = 4.4 × 10^−8^ M^2^ [80,81,82,83,84,85]). That is, Ag^+^ has the second highest affinity for I^−^, surpassed only by Hg_2_^2+^. In the presence of 10 μM of Ag^+^, for example, the activity of I^–^ in a solution cannot exceed 20 pM. Conversely, in the presence of 10 μM of Hg_2_^2+^, *a*_I_^−^ ≤ 6 pM, and for 10 μM of Pb^2+^ and Tl^+^, *a*_I_^−^ ≤ 21 and 4.4 mM, respectively. In addition, the p*K_sp_* of CuI is about 12, which makes Cu^+^ also a good iodine-ion “sponge.” The redox properties of copper (I) and the catalytic activity of some of its chelates make its oxide and salts prone to causing undesirable side reactions. While these *K_sp_* values are for the ion activities in aqueous media, they provide key guidelines showing that Ag^+^ is a halide “sponge,” almost as potent as Hg_2_^2+^, and considerably outperforms Tl^+^ and Pb^2+^. Similar to Ag(CH_3_CO_2_), the acetates of these two ions, Tl(CH_3_CO_2_) and Pb(CH_3_CO_2_)_2_, do not drive product formation. In contrast to Ag_2_O, their oxides, Tl_2_O and PbO also proved as inefficient as their salts, which can be ascribed to the affinity of Tl^+^ and Pb^2+^ for I^−^, which is an order to two-orders of magnitude smaller than that of Ag^+^.

While the *K_sp_* values offer good guidelines for the propensity of iodide formation, they do not truly represent the PIA reaction conditions. Most of the available information on the *K_sp_* of these halides is for an aqueous solution and not for the aprotic organic media that we employed for PIA. During the reaction, the dispersed solid phase in the mixture must convert from oxide to iodide. Examining the crystal-lattice properties of the different solid oxides and iodides revealed important trends that are strikingly consistent with the observed results for PIA. Ag_2_O and AgI are both polymorphic with their most stable structures assuming cubic lattice arrangements (Table 2). The oxide is slightly more stable than the iodide, but only by less than *2kBT* at room temperature, considering the most stable polymorphs of the two compounds (Table 2). This indicates the thermal plausibility of transforming Ag_2_O to AgI in the presence of iodide and proton sources to bind O^2−^ ions of the oxide and replace them with I^−^.

The porous structure of the cubic Ag_2_O (i.e., with the ions assuming face-centered antifluorite arrangement in the lattice) is quite beneficial for heterogeneous reactions. With a pore size of about 8 nm and specific surface area exceeding 9 m^2^ g^−1^, cubic Ag_2_O is an attractive material for heterogeneous catalysis [86]. While Ag_2_O is a reagent in PIA, rather than a catalyst, the accessibility of the starting materials from the liquid phase into its porous bulk is an important feature for this alkylation. In addition, the face-centered cubic AgI is a highly promising ion conductor [87]. This ion mobility in such materials is important for the replacement of O^2−^ with 2 I^−^ as the crystal lattice rearranges during the PIA reaction. Furthermore, AgI exhibits higher solubility in certain aprotic organic solvents than in aqueous media (i.e., *K_sp_*(AgI) is about two to four orders of magnitude smaller for acetonitrile and DMSO, respectively, than for water) [88]. While an increase in *K_sp_*(AgI) increases the thermodynamic driving force toward the products, in the case of heterogeneous reactions involving a solid starting material and a solid product, partial solubility of at least one of these solids can lower the energy of the transition states and the intermediates, which increases the reaction rates proving beneficial for the kinetics of the reaction. That is, partial desolation of the formed AgI in the organic liquid environment (especially, at elevated temperatures), prior to deposition on a solid phase, can aid the transformation from Ag_2_O to AgI.

Extending this thermodynamic line of thinking to Tl^+^ revealed that its oxide was more than 140 meV more stable than its iodide (Table 2), precluding a direct transformation of Tl_2_O into TlI. Initial dissolution of Tl_2_O in acidic solution, followed by treatment with a soluble metal halide, MX*_n_*, is required to form the corresponding TlX. Following this train of thought revealed that transforming the Pb^2+^ oxide into iodide is even more preposterously impossible than for Tl^+^, considering the energy of formation of PbO is 800 meV lower than that of PbI_2_ (Table 2).

These thermodynamic considerations feasibly outline the reasons why Ag_2_O led to 40% conversion of **1** into **3** and **4** (Figure 3), while Tl_2_O and PbO did not generate even traces of the products detectable via MS. In addition, it is important to consider possible intermediate structures that can affect the kinetics of transforming the oxides into iodides in aprotic organic media where dissolution of the inorganic phase is negligible to impossible. The stable polymorphs of Ag_2_O and AgI assume the form of the cubic lattices: not the same space groups, but still strikingly similar structures. Therefore, gradual transformation of silver oxide into its iodide does not require significant lattice rearrangements that may impose bindery energies that can be attributed to a decrease in the reaction rates. On the other hand, the significant lattice differences between Tl_2_O and TlI, and between PbO and PbI_2_ (Table 2), can induce further impedance on the oxide-to-iodide transformations, in addition to the thermodynamic infeasibility.

The solubility of the products and the lattice characteristics of the involved solids unequivocally show why Ag_2_O is such a good PIA reactant, while Tl_2_O and PbO are completely incapable of driving the reaction. However, what about mercury(I)? Among the iodides, Hg_2_I_2_ exhibits an impressively large p*K_sp_* of about 21 [80]. Tests with Hg_2_SO_4_ as a iodide-“sponge” reactant for PIA did not yield even traces of products. This is consistent with the enormous stability of the solid Hg_2_SO_4_ (monoclinic lattice), exhibiting Δ*Gf^(0)^* of about −1.45 eV per atom [79]. At the same time, Hg_2_O is unstable and disproportionate to HgO and Hg, making it an unfeasible candidate for a PIA reactant. The p*K_sp_* of HgI_2_, amounting to about 12, suggests the possible utility of the oxidatively stable Hg^2+^ as an iodide “sponge.” Nevertheless, the use of HgO for PIA is thermodynamically unfeasible: Δ*G_f_*^(0)^ of HgO (orthorhombic) and HgI_2_ (tetragonal) is −0.65 and 0.31 eV per atom, respectively [79].

All these considerations illustrate the uniqueness of Ag_2_O as an efficient scavenger of the hydrogen halides produced during etherification of alcohols with organic halides. Indeed, replacement of silver reagents with compounds comprising non-precious metal seems to be an important motivation in the search of alternative halide “sponges.” As the 67th and 68th for their abundance, Hg and Ag have practically the same rarity amounting to about 50 to 80 ppb of the Earth’s crust. Thallium is about 10 times more abundant than silver, but Tl^+^ has lesser affinity for halides than Ag^+^. As the 36th most abundant element, lead composes more than 10 ppm of the Earth’s crust, and from that point of view, it may present a feasible avenue to pursue in the search for alternative PIA reactants. Nevertheless, despite the 200 times planetary excess of lead over silver, toxicity and environmental considerations make silver by far the preferred choice.

Overall, the pronounced toxicity of mercury makes the pursuit of using its compounds for a reactant hugely unfeasible and impractical. Thallium and lead compounds are also immensely toxic. In contrast, silver compounds are some of the safest to use. After all, following the discovery of the photovoltaic effect with AgCl junction in the 19th century [89], AgBr was crucial for the development of photography [90,91]. Despite the toxicity of mercury, silver amalgams are a popular material for dental fillings. In fact, the United States Environmental Protection Agency’s recommended safety level of silver ions in drinking water (about 0.1 mg L^−1^) are almost the same as the admissible one of iron (about 0.3 mg L^−1^). Conversely, the amounts of mercury must not exceed 2 ppb, thallium must be less than 0.5 μg L^−1^, and lead must be below 15 μg L^−1^. These safety and environmental consideration provide a strong argument for the utility of silver compounds for etherification reactants. Fortunately, our findings unequivocally show that among the readily commercially available compounds of ions with potentially high affinity for halides, Ag_2_O is not only the best performing, but it is the only one that drives the etherification transformation (Table 2, Figure 3).

### 2.4. Microwave vs. Conventional Heating

For methylating carbohydrates in aqueous suspensions, PIA leads to high yields when carried out for extended periods of time. Lowering medium polarity increases the energy of the transition states, leading to a decrease in reaction rates. For PIA in non-polar media, heating is essential for ensuring the completion of the conversion in reasonable time. Employing microwave heating led to the maximum conversion of 40% in 1 min, producing an equimolar mixture of **3** and **4**. Transferring the microwave conditions to conventional heating in a pressure tube (i.e., 1 mmol of **1** and 2 mmol of Ag_2_O suspended in **2** and heated at 130 °C overnight) led to a 90% conversion to **4** with no traces of **3**. In comparison with the optimized base-driven etherification, PIA led to superbly higher yields (i.e., WE in 2ME in the presence of Cs_2_CO_3_ resulted in 40% transformation of **1** to **3** [27]), while PIA under conventional heating led to 90% conversion of **1** to **4**.

This drastic difference between the yields and the products from microwave and convectional heating reveals important features of the reaction (Figure 3). Under the former conditions, Ag_2_O is the principal absorber of the microwave radiation and it is heated considerably faster than the surrounding organic media. The short microwave bursts, along with the finite rates of heat transfer from the solid oxide to the liquid, make the solution at the surface of the Ag_2_O crystals hotter than that in the bulk of the organic phase. On the other hand, under conventional heating, the liquid phase is heated at the walls of the reaction vessel and transfers the heat to the solid oxide. Conversion of **1** to **4** is the first reaction step. Consequent hydrolysis of **4** leads to the formation of **3**. The considerably higher overall PIA yield for conventional rather than microwave heating suggests that the elevated temperature of the organic liquid is of primary importance for successful PIA, leading to etherification and esterification. Conversely, heat concentration on the basic solid oxide and at its interface with the organic reactants is essential for ester hydrolysis. These findings potentially have broad applicability in the design of heterogeneous synthetic procedures and the use of microwave heating only where it is most beneficial.

### 2.5. Box-Containing Oligomers

**Figure 4:**
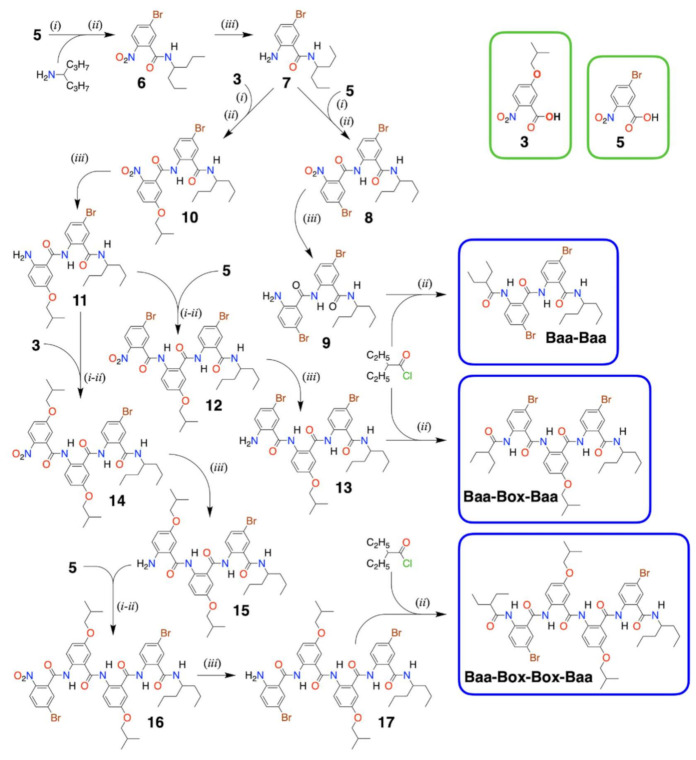
Synthesis of different bioinspired molecular electrets (in the blue frames) from two NBA precursors for Aa residues (i.e., **3** for Box and **5** for Baa (in the green frames)) employing (*i*) carboxylate activation, (*ii*) amid coupling, and (*iii*) nitro reduction; (*i*,*ii*) designates in situ activation (i.e., the carboxylate activation is carried out in the presence of the N-terminal amine). To attach NBA derivatives to a Baa N-terminal amine, chlorination activation (*i*) of the NBA carboxylates was carried out separately, prior to amid coupling (*ii*) (i.e., (1) NBA, (COCl)_2_, DCM, DMF, −78 °C to r.t., 1 h; (2) N-terminal amine, DCM, pyridine, −78 °C to r.t., overnight, 59% yield for **7** → **8** and 33% for **7** → **10**). To attach NBA derivatives to a Box N-terminal amine, carbodiimide carboxylate activation was carried out in situ with amid coupling (*i*,*ii*) (i.e., NBA, N-terminal amine, DIC, HOAt, DMAP, pyridine, DMF, r.t., overnight, 80% for **11** → **14** and 56% for **15** → **16**). Dicobalt octacarbonyl allows for selective reduction of the nitro groups (*iii*) (i.e., Co_2_(CO)_8_, 1,2-dimethoxyethane, 90 °C, 4 h; for N-terminal NO_2_ on Baa: 72% for **6** → **7**, 75% for **8** → **9** and 34% for **16** → **17**; and for N-terminal NO_2_ on Box: 33% for **10** → **11** and 47% for **14** → **15**). The results from HRMS, and ^1^H and ^13^C NMR analyses (Figures S3–S14), confirm the identities of the products.

With NBA derivatives in hand, the coupling/reduction protocol offers synthetic routes for bioinspired molecular electrets with diverse lengths (i.e., *n*) and sequence composition (i.e., R_1_ and R_2_ side chains on each of the Aa residues) (Figure 2). To demonstrate the utility of the ether NBA for composing oligomers with Box residues for which **3** is a precursor, we synthesized trimers and tetramers that also contained brominated Aa residues, Baa (Figure 4). 5-bromo-2-nitrobenzoic acid, **5**, is the NBA precursor for Baa. Baa residues are of outmost importance for post-synthetic modification of Aa molecular electrets. Homogeneous catalysis allows for replacement of the Br substituent with other functional groups. For example, catalytic replacement of Br with azide and its consequential reduction to amine provide sites for amide coupling of other moieties to the specific Baa sites of the electret sequence [25]. This strategy is essential for attaching potent electron acceptors to the Aa conjugates. Most electron acceptors and electron-deficient photosensitizers cannot sustain the reducing conditions required for converting the N-terminal nitro groups to amines before each coupling step. Building the Aa sequences with Baa residues allows for mild conversation of -Br to -NH_2_ in the synthesized oligomers, to which a carboxylate derivative of the electron-deficient moieties is readily attached [25].

Building the Aa oligomers comprising Baa and Box revealed important features about the coupling and the reduction steps in the protocol. For the selective reduction of the N-terminal nitro groups, we used dicobaltum octacarbonyl, Co_2_(CO)_8_ [92,93]. Using organic solutions of CrCl_2_ at room temperature offers the best yields for selective reduction of nitro groups to amines attached to Aa residues [29]. Nevertheless, the high sensitivity of CrCl_2_ to oxygen warrants carrying out such reduction reactions in an oxygen-free glove box, which renders their applicability for routine synthesis somewhat unfeasible. The relative kinetic stability of Co_2_(CO)_8_ provides an attractive alternative for employing it as a reducing agent. While the yields of converting -NO_2_ to -NH_2_ with Co_2_(CO)_8_ are relatively high (i.e., usually exceed 70%), introducing a Box residue in the sequence tends to lower them to about 30% to 50% (Figure 4, Scheme S2). Box is more electron-rich than Baa. Additionally, while Box manifests reversibility of its electrochemical oxidation at increased scan rates, the reduction potential of Box^•+^ exceeded 1.5 V vs. SCE [27], making it and its derivatives relatively susceptible to oxidative degradation. These electronic characteristics of ether Aa derivatives can render the formation of stable oligomers with amines on or near Box residues quite unfavorable.

The susceptibility of amines on N-terminal Box residues also governs the choice of amide coupling conditions. Activating the NBA precursors by converting them to acyl chlorides provides some of the most favorable conditions for building Aa oligomers [17,29]. For building Baa-Baa and Box-Baa sequence motifs, we chlorinated the acyl chlorides of **3** and **5** and added them to a cold solution of Baa with a free terminal amine, **7** (Figure 4). Similarly, we used aliphatic acyl chloride to cap the Baa N-terminal amines of **9**, **13**, and **17** (Figure 4). The same synthetic strategy, however, proved unfeasible for attaching the NBA derivatives **3** and **5** to the Box N-terminal amines of **11** and **15** (Figure 4).

Carboxyl chlorides are immensely strong acetylating agents. In addition, the small size of chloride and fluoride leaving groups makes these acyl halides immensely favorable for avoiding steric hindrance during amide coupling with the not-too electrophilic aromatic amines at the N-termini. The favored strong reactivity of acyl chlorides is also their demise. Even traces of moisture readily hydrolyze the chlorides to the corresponding carboxylic acid. Impurities of even weak and sterically hindered nucleophiles, formed from solvent degradation, for example, can lead to side products. Carboxylates activated as HOSu, HOBt, and HOAt esters, on the other hand, preferentially react with amine nucleophiles. Furthermore, the hydrogen-bonding propensity of the nitrogen in the benzene ring of HOAt can stabilize the transition states during amide coupling, making it a considerably more potent reagent than its HOBt analogue. Therefore, for amide coupling to Box N-terminal amines of **11** and **15**, we employed in situ carbodiimide activation in the presence of HOAt (Figure 4, Scheme S2).

These findings point out the underlying complexity in the design of synthetic strategies for making Aa molecular electrets (Figure 2). Electron-donating R_2_ substituents increase the electrophilicity of the N-terminal amine. The free amine on such electron-rich residues may readily increase their susceptibility to oxidative degradation. Conversely, hydrogen bonding with the carbonyl oxygen at the ortho position forces the N-terminal amine to be coplanar with the aromatic ring, which decreases its nucleophilicity. Hence, it is essential to select strong enough activation of the NBA carboxylate that undergoes amide coupling under mild enough conditions to prevent deuteriation of the amine starting material. Such optimal selection of activation and coupling strategies varies among the different sequence motifs of the Aa oligomers and warrants good understanding of the electronic properties of the comprising residues.

## 3. Conclusions

As promising as ether Aa residues are for hole-transfer bioinspired molecular electrets, their preparation is essential for exploring their utility and potential benefits. Robust and scalable methodologies for the synthesis of the ether derivatives of the 2-nitrobenzoic acid, NBA, precursors determine how accessible such electret conjugates can be. Is Ag_2_O -driven Purdie-Irvine alkylation the silver bullet for such synthetic goals? Considering the 90% overall conversion yield under solvent-free conditions and conventional heating in the presence of Ag_2_O, warrants a definite “yes” for an answer. The highest yields from reacting **1** and **2** under the conditions of optimized Williamson etherification were about 40% (Table 1). The Purdie-Irvine alkylation led to the formation of the ester of the desired ether derivative of the nitrobenzoic acid, warranting an additional hydrolysis step. The basic conditions of Williamson etherification ensure completion of the ester hydrolysis to produce the desired ether derivative of nitrobenzoic acid, but it does not lead to nearly as high yields as the Ag_2_O driven transformations. The close-to-quantitative yields that Purdie-Irvine reaction reveal important potential pathways for addressing the shortcomings of the Williamson etherification.

## Figures and Tables

**Table 1:** Optimization of silver-oxide loading for microwave reaction conditions.

***n*(Ag_2_O)/*n*(1) *^a^*^,*b*,*c*^**	**Yield of 3 *^d^***	**Yield of 4 *^d^***
1	9%	18%
1.5	10%	23%
2	20%	20%
**Previous studies: *^e^ n*(Cs_2_CO_3_)/*n*(1)**		
3	40%	0%

*^a^* 183 mg of **1** (1 mmol), 1 mL of **2** (8.7 mmol), microwave 60 W, 130 °C, time 2 × 30 s. *^b^* Molar excess of Ag_2_O in comparison with the NBA reactant **1**. *^c^* An increase in the excess of Ag_2_O to 2.5 and higher did not improve the yields. *^d^* Yields of isolated purified products. *^e^* 92 mg of **1** (0.5 mmol), 0.23 mL of **2** (2 mmol), 1 mL 2ME, microwave 60 W, 130 °C, time 2 × 30 s [27].

**Table 2:** Characteristics of the oxides and iodides of silver(I), thallium(I), and lead(II) [79].

Oxide or Iodide	Crystal Lattice *^a^*	Δ*G_f_*^(0)^*/*eV per Atom *^b^*	*ρ/*g cm^−3 *c*^	Yield*/% ^d^*
Ag_2_O	cubic (m$\bar{3}$m, Pn$\bar{3}$m [224]), stable	−0.328	6.78	40 (90) ^*e*^
	triclinic (1, P1 [1])	−0.219	6.87	
	trigonal (m$\bar{3}$m, P$\bar{3}$m1 [164])	−0.208	8.61	
AgI	cubic ($\bar{4}$3m, F$\bar{4}$3m [216]), stable	−0.281	5.32	—
	cubic (m$\bar{3}$m, Fm$\bar{3}$m [225])	−0.188	6.64	
	cubic (m$\bar{3}$m, Pm$\bar{3}$m [221])	−0.109	6.80	
	hexagonal (6mm, P63mc [186])	−0.280	5.33	
	hexagonal (6mm, P63mc [186])	−0.234	5.29	
	tetragonal ($\bar{4}$2m, I$\bar{4}$m2 [119])	−0.279	5.32	
	tetragonal (4/mmm, P4/nmm [129])	−0.256	5.55	
	monoclinic (2/m, P21/m [11])	−0.177	6.69	
Tl_2_O	trigonal ($\bar{3}$m, R$\bar{3}$m [166]) stable	−0.826	9.41	0
TlI	cubic (m$\bar{3}$m, Fm$\bar{3}$m [225]) stable	−0.682	6.05	—
	cubic (m$\bar{3}$m, Pm$\bar{3}$m [221])	−0.629	6.98	
	orthorhombic (mmm, Cmcm [63])	−0.658	6.55	
PbO	orthorhombic (mmm, Pbcm [57]) stable	−1.477	8.31	0
	orthorhombic (mmm, Pbcm [57])	−1.458	8.74	
	orthorhombic (mm2, Pca21 [29])	−1.456	7.89	
	tetragonal (4/mmm, P4/nmm [129])	−1.476	8.47	
	tetragonal (4/mmm, P42/mmc [131])	−1.064	8.37	
PbI_2_	hexagonal (6mm, P63mc [186]) stable	−0.668	5.12	—
	hexagonal (6mm, P63mc [186])	−0.668	5.13	
	trigonal (3m, P3m1 [156])	−0.668	5.34	
	trigonal ($\bar{3}$m, P$\bar{3}$m1 [164])	−0.667	5.36	
	trigonal ($\bar{3}$m, R$\bar{3}$m1 [166])	−0.668	5.11	
	trigonal ($\bar{3}$m, R$\bar{3}$m1 [166])	−0.666	5.34	

*^a^* Crystal system (point group, Hermann-Mauguin notation with the corresponding [space-group number]). For each compound, “stable” designates the most stable structures to which other ones may rearrange. *^b^* Formation energy. *^c^* Mass density. *^d^*Yields of conversion of **1** to **3** and **4** under microwave heating. *^e^* The value in the parentheses represents the yield under conventional heating.

## Data Availability

Not applicable.

## Supplementary Materials

The following are available online at https://www.mdpi.com/2218-273X/11/3/429/s1, Scheme S1.: Alkylation of 5-hydroxy-2-nitrobenzoic acid leading to its ether and ester derivatives; Scheme S2.: Synthesis of anthranilamide oligomers; Figures S1 to S14: (a) ^1^H NMR of **X** (600 MHz, CDCl_3_); (b) ^13^C NMR of (X) (151 MHz, CDCl_3_), where **X** = **3**, **4**, **6**, **7**, **9**, **Baa-Baa**, **11**, **12**, **13**, **Baa-Box-Baa**, **14**, **15**, **16**, **Baa-Box-Box-Baa**.
