# Supplementary material for: On the Search of a Silver Bullet for the Preparation of Bioinspired Molecular Electrets with Propensity to Transfer Holes at High Potentials
Source: Biomolecules. 2021 Mar 15;11(3):429. doi: 10.3390/biom11030429 (PMC8001849; doi:10.3390/biom11030429)
Supplement: Supplementary file 1 [file biomolecules-11-00429-s001.pdf]

On the Search of a Silver Bullet for the Preparation of Bioinspired Molecular Electrets with Propensity to Transfer Holes at High Potentials (Supplementary Materials)

James Bennett Derr [1], Katarzyna Rybicka-Jasińska [2,†], Eli Misael Espinoza [3,‡], Maryann Morales [3,§], Mimi Karen Billones [4], John Anthony Clark [2] and Valentine Ivanov Vullev [1,2,3,5,*]

[1] Department of Biochemistry, University of California, Riverside, CA 92521, USA; jderr002@ucr.edu
[2] Department of Bioengineering, University of California, Riverside, CA 92521, USA; katarzyna.rybickajasinska@gmail.com (K.R.-J.); jclar019@ucr.edu (J.A.C.)
[3] Department of Chemistry, University of California, Riverside, CA 92521, USA; eespi009@berkeley.edu (E.M.E.); mmorale2@caltech.edu (M.M.)
[4] Department of Biology, University of California, Riverside, CA 92521, USA; mbill010@ucr.edu
[5] Department of Materials Science and Engineering Program, University of California, Riverside, CA 92521, USA
* Correspondence: vullev@ucr.edu; Tel.: +1-(951)-827-6239
† Present address: Institute of Organic Chemistry, Polish Academy of Sciences, Warsaw 01-224, Poland
‡ Present address: College of Bioengineering, University of California, Berkeley, CA 94720, USA
§ Present address: Division of Chemistry and Chemical Engineering, California Institute of Technology, Pasadena, CA 91125, USA

General methods. All chemicals were used as received unless otherwise specified. The reported ^{1}H NMR, ^{13}C NMR, and NOESY spectra were recorded on 400 MHz, 500 MHz and 600 MHz spectrometers. ^{1}H chemical shifts (δ) are reported in ppm relative to $CHCl_3$ in $CDCl_3$ (δ = 7.26 ppm); ^{13}C δ are reported in ppm relative to $CDCl_3$ (δ = 77.23 ppm). Data for ^{1}H NMR are reported as follows: chemical shift, integration, multiplicity (s = singlet, d = doublet, t = triplet, q = quartet, p = pentet/quintet, h = hextet/sextet, e = eptet(from επτά)/heptet, m = multiplet), and coupling constants. All ^{13}C NMR spectra were recorded with complete proton decoupling. High-resolution mass spectrometry (HRMS) was performed using Agilent LCTOF (6200) mass spectrometer (Agilent Technologies, Santa Clara, CA). Analytical thin layer chromatography was performed using 0.25 mm silica gel 60-F plates. Flash chromatography was performed using 60 Å, 32−63 μm silica gel.

Synthetic procedures

Scheme S1. Alkylation of 5-hydroxy-2-nitrobenzoic acid leading to its ether and ester derivatives.

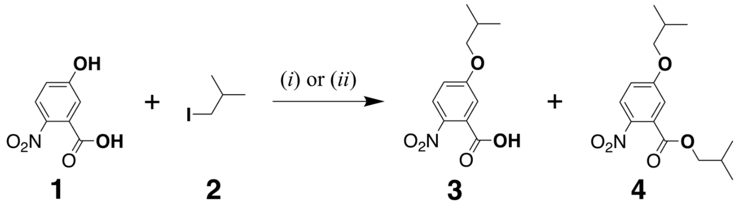

(*i*) C_4H_9I, Ag_2O, 130°C, 60 sec, 20% **(2),** 20% **(3)**; (*ii*) C_4H_9I, Ag_2O, 130°C, 18 h, 90% **(3)**.

Scheme S2. Synthesis of anthranilamide oligomers.

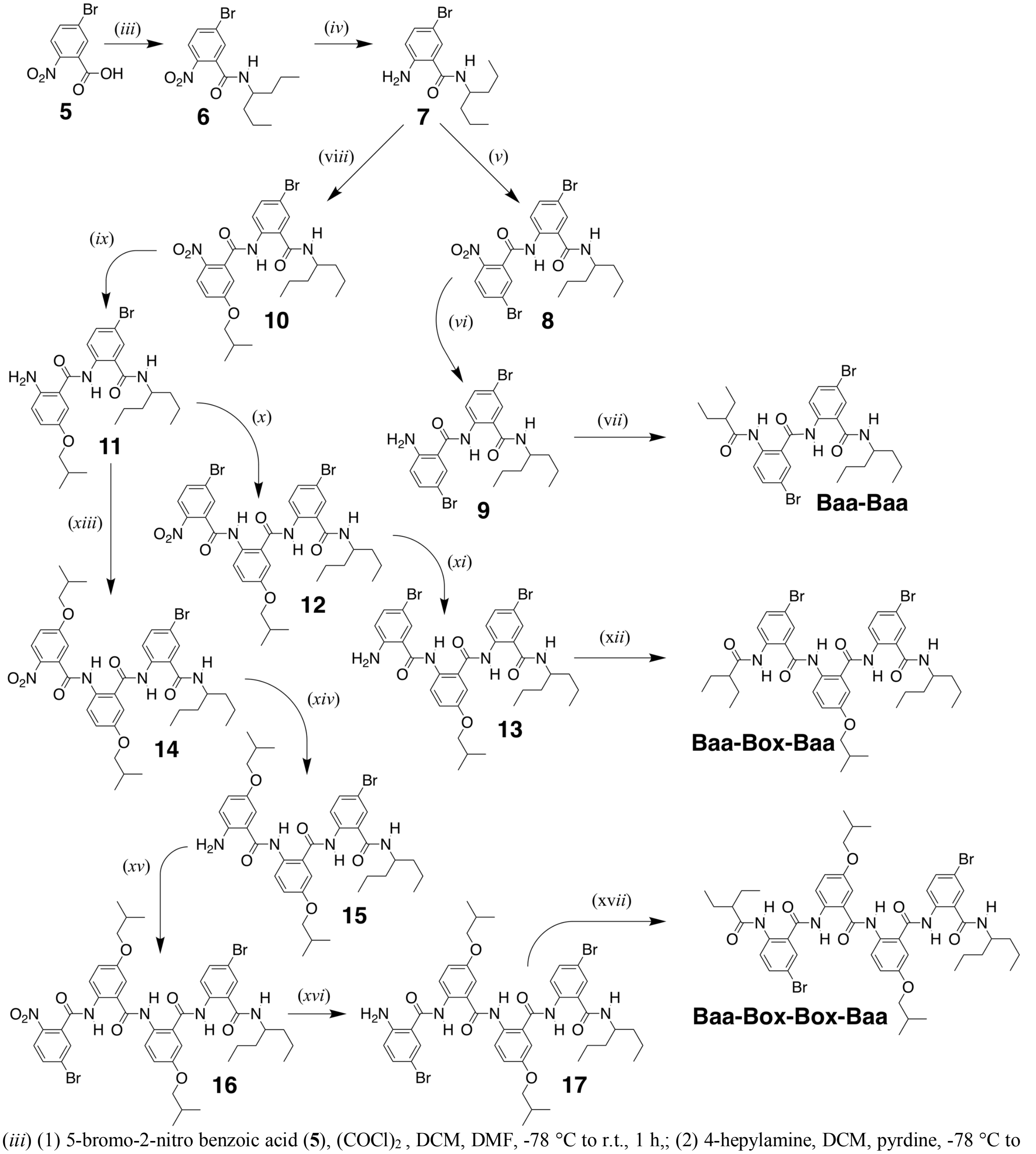

(*iii*) (1) 5-bromo-2-nitro benzoic acid (**5**), $(COCl)_2$, DCM, DMF, -78 °C to r.t., 1 h,; (2) 4-hepylamine, DCM, pyrdine, -78 °C to r.t., overnight, 92%; (*iv*) $Co_2(CO)_8$, 1,2-Dimethoxyethane, 90°C, 4 h, 72%; (*v*) (1) **5**, $(COCl)_2$, DCM, DMF, -78 °C to r.t., 1 h,; (2) DCM, pyridine, -78 °C to r.t., overnight, 59%; (*vi*) $Co_2(CO)_8$, 1,2-Dimethoxyethane, 90°C, 4 h, 75%; (*vii*) 2-ethylbutyryl chloride, pyridine, THF, r.t., 1 h, 100%; (*viii*) (1) **3**, $(COCl)_2$, DCM, DMF, -78 °C to r.t., 1 h; (2) DCM, pyridine, -78 °C to r.t., overnight, 0.57%; (*ix*) $Co_2(CO)_8$, 1,2-Dimethoxyethane, 90°C, 4 h, 33%; (*x*) **5**, DIC, HOAt, DMAP, pyridine, DMF, r.t., Overnight, 80%; (*xi*) $Co_2(CO)_8$, 1,2-Dimethoxyethane, 90°C, 4 h, 61%; (*xii*) 2-ethylbutyryl chloride, pyridine, THF, r.t., 1 h, 85%; (*xiii*) **3**, DIC, HOAt, DMAP, pyridine, DMF, r.t., Overnight, 95%; (*xiv*) $Co_2(CO)_8$, 1,2-Dimethoxyethane, 90°C, 4 h, 47%; (*xv*) **5**, DIC, HOAt, DMAP, pyridine, DMF, r.t., Overnight, 56%; (*xvi*) $Co_2(CO)_8$, 1,2-Dimethoxyethane, 90°C, 4 h, 34%; (*xvii*) 2-ethylbutyryl chloride, pyridine, THF, r.t., 1 h, 58%

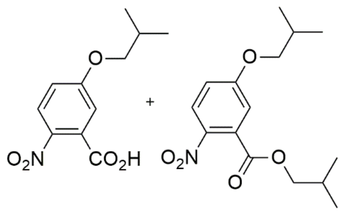

5-isobutoxy-2-nitrobenzoic acid (3) and isobutyl 5-isobutoxy-2-nitrobenzoate (4) (Scheme S1). ***Microwave-heating procedure.*** Ag_2O (463 mg, 2 mmol) was placed in a microwave vial, and purged with argon. Then 5-hydroxy-2-nitrobenzoic acid (**1**) (183 mg, 1 mmol). was added, followed by the 1-iodo-2-methylpropane (**2**) (1 mL). The microwave vial was capped and put into the microwave. The parameters were set to 130°C, 60 W, 2×30 seconds. After the first interval the reaction mixture was allowed to cool for 5 minutes and then microwaved again at the same exact parameters giving a dark orange solution. The progress of the reaction was monitored via TLC. The mixture was diluted with 5% HCL and extracted with DCM (3×25mL). The organic layer was dried Na_2SO_4 and condensed. The products was purified using flash chromatography (stationary phase: silica gel; eluent gradient: 0:1 (v:v) to 1:1 (v:v) of ethyl acetate and hexanes. The 1:1 elution was mixed with 1% acetic acid. The product was condensed to afford 47 mg (20%) of **3** (white crystals) and 60 mg (20%) of **4** (brown oil). For **3**: ^{1}H NMR (600 MHz, Chloroform-*d*) δ 8.0khggi 2 (d, J = 9.1 Hz, 1H), 7.17 (d, J = 2.7 Hz, 1H), 7.05 (dd, J = 9.1, 2.7 Hz, 1H), 3.84 (d, J = 6.5 Hz, 2H), 2.13 (dq, J = 13.3, 6.7 Hz, 1H), 1.05 (d, J = 6.7 Hz, 6H) ppm. ^{13}C NMR (151 MHz, $CDCl_3$) δ 163.3, 140.1, 130.3, 126.9, 116.9, 115.0, 77.4, 77.2, 77.0, 75.7, 29.9, 28.3, 19.3, 19.1 ppm. HRMS (ESI) m/z calculated for $C_{11}H_{13}NO_5$: $[M-H]^-$ 238.0721, found 238.0827. For **4**: ^{1}H NMR (600 MHz, Chloroform-d) δ 7.98 (d, J = 9.0 Hz, 1H), 7.02 (d, J = 2.7 Hz, 1H), 6.98 (dd, J = 9.0, 2.8 Hz, 1H), 4.09 (d, J = 6.6 Hz, 2H), 3.79 (dd, J = 6.5, 3.4 Hz, 2H), 2.14 – 2.05 (m, 1H), 2.01 (m, J = 13.4, 6.7 Hz, 1H), 1.01 (d, J = 6.7 Hz, 6H), 0.94 (d, J = 6.7 Hz, 6H) ppm. ^{13}C NMR (151 MHz, $CDCl_3$) δ 166.4, 163.2, 139.9, 131.6, 126.8, 126.6, 115.9, 114.8, 77.4, 77.2, 77.0, 75.5, 72.8, 28.2, 27.9, 27.7, 19.2, 19.2 ppm. HRMS (ESI) m/z calculated for $C_{15}H_{21}NO_5$: $[M+H]^+$ 295.1420, found 296.1491.

Conventional-heating procedure. 5-hydroxy-2-nitrobenzoic acid **(1)** (183 mg, 1 mmol), was placed in in a pressure tube with a stir bar and purged with argon. Two mL of 1-iodo-2-methylpropane (**2**) was added, followed by the Ag_2O (463 mg, 2 mmol. The mixture was stirred at 130 °C overnight. The progress of the reaction was monitored via TLC. The reaction was taken out of the oil bath for 10 minutes to cool and then diluted with 200 mL of 5% HCl and extracted with DCM (3×25mL). Dried over Na_2SO_4 and vacuum filtered. The filtrate was condensed. The product was purified using flash chromatography (stationary phase: silica gel; eluent gradient: 0:1 (v:v) to 1:1 (v:v) of ethyl acetate and hexanes. The 1:1 elution was mixed with 1% acetic acid. The product was condensed to afford 267 mg (90%) of **(3)** as a brown oil.

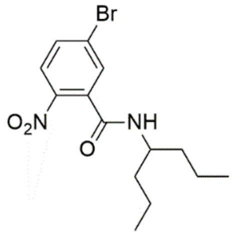

5-bromo-N-(heptan-4-yl)-2-nitrobenzamide (6) (Scheme S2). 5 (500 mg, 2.03 mmol) was transferred to a dry, Ar purged 100 mL round bottom flask, with a stir bar. This was dissolved in 20 mL of DCM, with 3 drops of DMF. The reaction mixture was cooled to -78°C and was stirred for 5 minutes. Then, (270 µL, 3.05 mmol) of oxalyl chloride was added dropwise, the stirred for 1 h. The reaction was monitored via TLC by taking a few drops of reaction mixture and adding it to methanol. This would generate the ester from and move up the TLC plate. The reaction mixture was condensed 3 times, each time adding 20 mL of DCM. The condensed product was dissolved in 20 mL of DCM and cooled to -78°C for 5 min. 4-heptylamine (550 µL, 3.65 mmol) was added dropwise followed by pyridine (250 µL, 3.05 mmol). This was raised to room temperature and reacted overnight. Upon completion of the reaction, the mixture was quenched with 50 mL of 5% HCl. The mixture was extracted with DCM (3×25mL). The organic layer was dried Na_2SO_4 and condensed. The product was purified using flash chromatography (stationary phase: silica gel; eluent gradient: 1:9 (v:v) to 1:4 (v:v) of ethyl acetate and hexanes. The product was condensed to afford 638 mg (92%) of **6** as a light yellow powder. ^{1}H NMR (600 MHz, Chloroform-*d*) δ 7.93 (d, J = 8.7 Hz, 1H), 7.68 (dd, J = 8.7, 2.2 Hz, 1H), 7.59 (d, J = 2.1 Hz, 1H), 5.55 (d, J = 9.2 Hz, 1H), 4.16 – 4.07 (m, 1H), 1.56 (m, J = 13.3, 5.0, 3.1, 1.9 Hz, 4H), 1.51 – 1.38 (m, 4H), 0.97 (t, J = 7.1 Hz, 6H) ppm. ^{13}C NMR (151 MHz, $CDCl_3$) δ 164.7, 145.2, 135.2,

133.4, 131.9, 128.8, 126.2, 77.4, 77.2, 77.0, 50.2, 37.2, 19.3, 14.3 ppm. HRMS (ESI) m/z calculated for $C_{14}H_{19}BrN_2O_3$: $[M+H]^+$ 342.0573 found 343.0647.

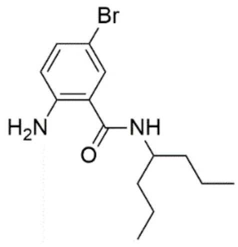

2-amino-5-bromo-N-(heptan-4-yl)benzamide (7) (Scheme S2). 6 (300 mg, 0.88 mmol) and $Co_2(CO)_8$ (600 mg, 1.75 mmol) were placed in a 25-mL pressure tube with a magnetic stir bar in it. While purging with argon, 10 mL of 1,2-dimethoxyethane (DME) and five drops of DI water was added to the tube and tightly closed. While mixing, the pressure tube was immersed in a temperature-controlled oil bath. The mixture was heated to 90 °C and stirred for four hours. It was taken out of the oil bath and allowed to cool to room temperature prior to opening it. The reaction mixture was filtered; the filtrate was collected and condensed then diluted with 20 mL DCM, and washed with water (100 mL). The organic layer was collected, dried over Na_2SO_4, and concentrated in vacuo. The product was purified using flash chromatography (stationary phase: silica gel; eluent gradient: from 1:9 (v:v) to 1:4 ratio of ethyl acetate and hexanes) to afford 198 mg (72%) of **7** as a white solid. ^1H NMR (600 MHz, Chloroform-*d*) δ 7.37 (d, J = 2.2 Hz, 1H), 7.29 (dd, J = 8.7, 2.3 Hz, 1H), 6.65 (d, J = 8.7 Hz, 1H), 5.96 (s, 2H), 5.65 (d, J = 9.1 Hz, 1H), 4.10 (qd, J = 8.3, 4.1 Hz, 1H), 1.60 – 1.51 (m, 2H), 1.49 – 1.32 (m, 6H), 0.94 (t, J = 7.2 Hz, 6H) ppm. ^{13}C NMR (151 MHz, $CDCl_3$) δ 167.7, 146.7, 134.9, 129.5, 119.6, 119.0, 108.9, 77.4, 77.2, 77.0, 49.3, 37.8, 19.4, 14.3 ppm. HRMS (ESI) m/z calculated for $C_{14}H_{21}BrN_2O$: $[M+H]^+$ 312.0836 found 313.0909.

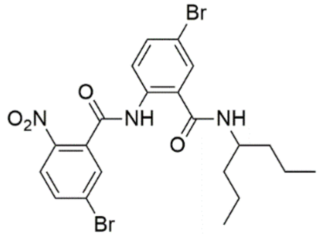

5-bromo-N-(4-bromo-2-(heptan-4-ylcarbamoyl)phenyl)-2-nitrobenzamide (8) (Scheme S2). 5 (244 mg, 0.99 mmol), was transferred to a dry, Ar purged 100 mL round bottom flask, with a stir bar. This was dissolved in 20 mL of DMF. The reaction mixture was cooled to -78°C and was stirred for 5 minutes. Then, (170 µL, 1.99 mmol) of oxalyl chloride was added dropwise, the stirred for 1 h. The reaction was monitored via TLC by taking a few drops of reaction mixture and adding it to methanol. This would generate the ester from and move up the TLC plate. The reaction mixture was condensed 3 times, each time adding 20 mL of DCM. The condensed product was dissolved in 20 mL of DCM and cooled to -78°C for 5 min. **7** (465 mg, 1.49 mmol) was added dropwise followed by pyridine (122 µL, 1.49 mmol). This was raised to room temperature and reacted overnight. Upon completion of the reaction, the mixture was quenched with 50 mL of 5% HCl. The mixture was extracted with DCM (3×25mL). The organic layer was dried Na_2SO_4 and condensed. The product was purified using flash chromatography (stationary phase: silica gel; eluent gradient: 0:1 (v:v) to 1:9 (v:v) of ethyl acetate and hexanes. The product was condensed to afford 312 mg (59%) of **8** as a yellow oil. HRMS (ESI) m/z calculated for $C_{21}H_{23}Br_2N_3O_4$: $[M+H]^+$ 539.0035 found 542.0092.

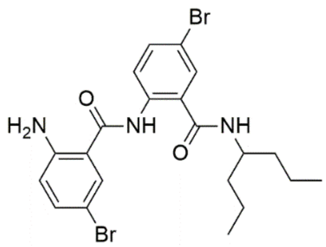

2-amino-5-bromo-N-(4-bromo-2-(heptan-4-ylcarbamoyl)phenyl)benzamide (9) (Scheme S2). 8 (212 mg, 0.39 mmol) and $Co_2(CO)_8$ (267 mg, 0.78 mmol) were placed in a 25-mL pressure tube with a magnetic stir bar in it. While purging with argon, 10 mL of 1,2-dimethoxyethane (DME) and five drops of DI water was added to the tube and tightly closed. While mixing, the pressure tube was immersed in a temperature-controlled oil bath. The mixture was heated to 90 °C and stirred for 4 hours. It was taken out of the oil bath and allowed to cool to room temperature prior to opening it. The reaction mixture was filtered; the filtrate was collected and condensed then diluted with 20 mL DCM, and washed with water (100 mL). The organic layer was collected, dried over Na_2SO_4, and concentrated in vacuo. The product was purified using flash chromatography (stationary

phase: silica gel; eluent gradient: from 0:1 (v:v) to 1:9 ratio of ethyl acetate and hexanes) to afford 149 mg (75%) of **9** as a yellow oil. ^{1}H NMR (600 MHz, Chloroform-*d*) δ 11.67 (s, 1H), 8.55 (d, *J* = 8.9 Hz, 1H), 7.72 (d, *J* = 2.3 Hz, 1H), 7.60 (dd, *J* = 8.9, 2.3 Hz, 1H), 7.55 (d, *J* = 2.3 Hz, 1H), 7.36 – 7.27 (d, 1H), 6.58 (dd, *J* = 12.1, 8.7 Hz, 1H), 5.83 (d, *J* = 9.2 Hz, 2H), 4.23 – 4.15 (m, 1H), 1.67 – 1.51 (m, 2H), 1.51 – 1.30 (m, 6H), 0.95 (t, *J* = 12.6, 7.2 Hz, 6H) ppm. ^{13}C NMR (151 MHz, $CDCl_3$) δ 167.6, 167.0, 148.8, 138.7, 135.8, 135.2, 130.6, 129.2, 123.7, 123.6, 119.2, 117.3, 115.6, 108.3, 77.4, 77.23, 77.0, 49.8, 37.7, 19.5, 19.4, 14.2, 1.2 ppm. HRMS (ESI) m/z calculated for $C_{21}H_{25}Br_2N_3O_2$: $[M+H]^+$ 509.0313 found 512.0370.

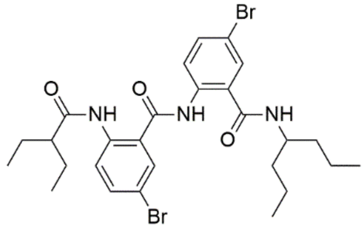

5-bromo-2-(5-bromo-2-(2-ethylbutanamido)benzamido)-N-(heptan-4-yl)benzamide (Baa-Baa) (Scheme S2). **9** (100 mg, 0.196 mmol) was transferred to a dry, Ar purged 100 mL round bottom flask, with a stir bar. This was dissolved in 20 mL of THF. The reaction mixture was cooled to -78°C and was stirred for 5 minutes. Then, (33 μL, 0.24 mmol) of 2-ethylbutyryl chloride was added dropwise followed by pyridine (80 μL, 0.98 mmol) and the stirred for 1 h. Upon completion of the reaction, the mixture was quenched with 50 mL of 5% HCl. The mixture was extracted with DCM (3×25mL). The organic layer was dried Na_2SO_4 and condensed. The product was purified using flash chromatography (stationary phase: silica gel; eluent gradient: 0:1 (v:v) to 1:9 (v:v) of ethyl acetate and hexanes. The product was condensed to afford 127 mg (100%) of **Baa-Baa** as a white solid. ^{1}H NMR (600 MHz, Chloroform-*d*) δ 12.08 (s, 1H), 11.12 (s, 1H), 8.67 (d, *J* = 9.0 Hz, 1H), 8.55 (d, *J* = 8.9 Hz, 1H), 7.89 (d, *J* = 2.3 Hz, 1H), 7.64 (dd, *J* = 8.9, 2.3 Hz, 1H), 7.62 – 7.58 (m, 2H), 5.90 (d, *J* = 9.2 Hz, 1H), 4.20 (qt, *J* = 8.9, 5.1 Hz, 1H), 2.19 – 2.11 (m, 1H), 1.77 – 1.64 (m, 2H), 1.64 – 1.54 (m, 4H), 1.53 – 1.34 (m, 6H), 0.95 (t, *J* = 7.2 Hz, 12H) ppm. ^{13}C NMR (151 MHz, $CDCl_3$) δ 181.6, 175.4, 167.4, 166.7, 139.6, 138.1, 136.1, 135.4, 130.3, 129.3, 123.7, 123.3, 122.0, 116.5, 115.6, 77.4, 77.2, 77.0, 53.1, 50.0, 48.7, 37.7, 25.9, 25.0, 19.5, 14.2, 12.2, 12.0 ppm. HRMS (ESI) m/z calculated for $C_{27}H_{35}Br_2N_3O_3$: $[M+Na]^+$ 607.1046 found 632.0932.

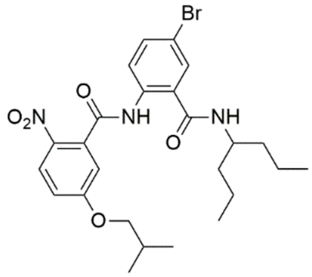

5-bromo-N-(heptan-4-yl)-2-(5-isobutoxy-2-nitrobenzamido)benzamide (10) (Scheme S2). **3** (1271 mg, 5.34 mmol) was transferred to a dry, Ar purged 100 mL round bottom flask, with a stir bar. This was dissolved in 40 mL of DCM, with 5 drops of DMF. The reaction mixture was cooled to -78°C and was stirred for 5 minutes. Then, (916 μL, 10.68 mmol) of oxalyl chloride was added dropwise, the stirred for 1 h. The reaction was monitored via TLC by taking a few drops of reaction mixture and adding it to methanol. This would generate the ester from and move up the TLC plate. The reaction mixture was condensed 3 times, each time adding 20 mL of DCM. The condensed product was dissolved in 20 mL of DCM and cooled to -78°C for 5 min. **6** (1834 mg, 5.88 mmol) in 10 mL of DCM was added dropwise followed by pyridine (650 μL, 8.01 mmol). This was raised to room temperature and reacted overnight. Upon completion of the reaction, the mixture was quenched with 50 mL of 5% HCl. The mixture was extracted with DCM (3×25mL). The organic layer was dried Na_2SO_4 and condensed. The product was purified using flash chromatography (stationary phase: silica gel; eluent gradient: 0:1 (v:v) to 1:9 (v:v) of ethyl acetate and hexanes. The product was condensed to afford 161.46 mg (0.57%) of **10** as a light yellow oil. HRMS (ESI) m/z calculated for $C_{25}H_{32}BrN_3O_5$: $[M+Na]^+$ 533.1530 found 558.1409.

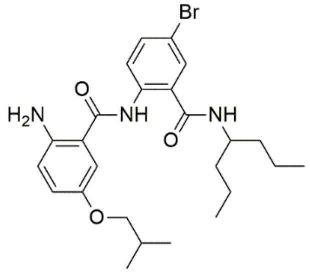

2-amino-N-(4-bromo-2-(heptan-4-ylcarbamoyl)phenyl)-5-isobutoxybenzamide (11) (Scheme S2). 10 (100 mg, 0.188 mmol) and $Co_2(CO)_8$ (128 mg, 0.375 mmol) were placed in a 25-mL pressure tube with a magnetic stir bar in it. While purging with argon, 10 mL of 1,2-dimethoxyethane (DME) and five drops of DI water was added to the tube and tightly closed. While mixing, the pressure tube was immersed in a temperature-controlled oil bath. The mixture was heated to 90 °C and stirred for four hours. It was taken out of the oil bath and allowed to cool to room temperature prior to opening it. The reaction mixture was filtered; the filtrate was collected and condensed then diluted with 20 mL DCM, and washed with water (100 mL). The organic layer was collected, dried over Na_2SO_4, and concentrated in vacuo. The product was purified using flash chromatography (stationary phase: silica gel; eluent gradient: from 0:1 (v:v) to 1:9 ratio of ethyl acetate and hexanes) to afford 31 mg (33%) of **11** as a dark yellow solid. ^{1}H NMR (600 MHz, Chloroform-*d*) δ 11.83 (s, 1H), 8.66 (d, *J* = 8.8 Hz, 1H), 7.65 – 7.58 (m, 2H), 7.22 (d, *J* = 2.8 Hz, 1H), 6.96 (dd, *J* = 8.8, 2.7 Hz, 1H), 6.69 (d, *J* = 8.8 Hz, 1H), 5.96 (d, *J* = 9.1 Hz, 1H), 5.15 (s, 2H), 4.21 – 4.14 (m, 1H), 3.78 (d, *J* = 6.6 Hz, 2H), 2.11 (m, *J* = 14.9, 7.5 Hz, 1H), 1.64 – 1.55 (m, 2H), 1.54 – 1.33 (m, 6H), 1.06 (d, *J* = 6.7 Hz, 6H), 0.96 (t, *J* = 7.3 Hz, 6H) ppm. ^{13}C NMR (151 MHz, $CDCl_3$) δ 167.9, 167.6, 151.2, 143.7, 139.1, 135.2, 129.2, 123.4, 123.1, 122.5, 119.4, 116.0, 115.1, 111.8, 77.4, 77.2, 77.0, 75.5, 49.7, 37.6, 28.5, 19.5, 19.5, 14.2 ppm. HRMS (ESI) m/z calculated for $C_{25}H_{34}BrN_3O_3$: $[M-H]^-$ 503.1820 found 502.1749.

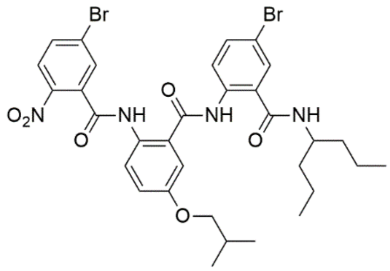

5-bromo-N-(2-((4-bromo-2-(heptan-4-ylcarbamoyl)phenyl)carbamoyl)-4-isobutoxyphenyl)-2-nitrobenzamide (12) (Scheme S2). 5 (787 mg, 3.2 mmol), 1-hydroxy-7-azabenzotriazole (HOAt) (490 mg, 3.6 mmol), N,N′-Diisopropylcarbodiimide (DIC) (500 µL, 3.2 mmol), 4-Dimethylaminopyridine (DMAP) (10 mg, 9.47 mmol) and pyridine (460 µL, 5.6 mmol) was dissolved in 20 mL of DMF in a dry, argon purged 100 mL round bottom flask with a stir bar. This was stirred for 15 minutes and then **11** (404 mg, 0.802 mmol) in 10 mL of DCM, was transferred and stirred overnight. The progress of the reaction was monitored via TLC. The mixture quenched with 100 mL of 5% HCl then was extracted with DCM (3×25mL). The organic layer was dried Na_2SO_4 and condensed. The product was purified using flash chromatography (stationary phase: silica gel; eluent gradient: 0:1 (v:v) to 1:9 (v:v) of ethyl acetate and hexanes. This afforded 471 mg (80%) of **12** as a yellow solid. ^{1}H NMR (600 MHz, Chloroform-*d*) δ 12.25 (s, 1H), 11.50 (s, 1H), 8.61 (d, *J* = 9.1 Hz, 1H), 8.52 – 8.47 (d, 1H), 7.97 (d, *J* = 8.7 Hz, 1H), 7.82 (d, *J* = 2.3 Hz, 1H), 7.74 (dd, *J* = 8.7, 2.1 Hz, 1H), 7.59 (d, *J* = 7.7 Hz, 2H), 7.39 (d, *J* = 2.8 Hz, 1H), 7.15 (dd, *J* = 9.1, 2.8 Hz, 1H), 5.92 (d, *J* = 9.1 Hz, 1H), 4.20 – 4.10 (m, 1H), 3.84 (d, *J* = 6.6 Hz, 2H), 2.14 (hept, *J* = 6.7 Hz, 1H), 1.63 – 1.55 (m, 2H), 1.52 – 1.33 (m, 6H), 1.06 (d, *J* = 6.7 Hz, 6H), 0.94 (t, *J* = 7.3 Hz, 6H) ppm. ^{13}C NMR (151 MHz, $CDCl_3$) δ 167.4, 162.7, 155.8, 145.6, 138.3, 135.4, 135.1, 133.8, 133.1, 131.9, 129.3, 129.0, 126.4, 123.7, 123.5, 123.3, 121.8, 120.5, 116.3, 112.5, 77.4, 77.2, 77.0, 75.1, 49.9, 37.5, 29.9, 28.4, 19.5, 19.4, 14.2 ppm. HRMS (ESI) m/z calculated for $C_{32}H_{36}Br_2N_4O_6$: $[M+Na]^+$ 730.0944, found 732.0921.

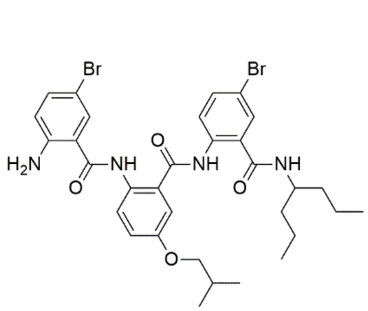

2-amino-5-bromo-N-(2-((4-bromo-2-(heptan-4-ylcarbamoyl)phenyl)carbamoyl)-4-isobutoxyphenyl)benzamide (13) (Scheme S2). 12 (362 mg, 0.496 mmol) and $Co_2(CO)_8$ (339 mg, 0.99 mmol) were placed in a 25-mL pressure tube with a magnetic stir bar in it. While purging with argon, 10 mL of 1,2-dimethoxyethane (DME) and five drops of DI water was added to the tube and tightly closed. While mixing, the pressure tube was immersed in a temperature-controlled oil

bath. The mixture was heated to 90 °C and stirred for 4 h. It was taken out of the oil bath and allowed to cool to room temperature prior to opening it. The reaction mixture was filtered; the filtrate was collected and condensed then diluted with 20 mL DCM, and washed with water (100 mL). The organic layer was collected, dried over Na_2SO_4, and concentrated in vacuo. The product was purified using flash chromatography (stationary phase: silica gel; eluent gradient: from 0:1 (v:v) to 1:9 ratio of ethyl acetate and hexanes) to afford 212 mg (61%) of **13** as a light yellow solid. ^{1}H NMR (600 MHz, Chloroform-*d*) δ 12.22 (s, 1H), 11.66 (s, 1H), 8.71 (d, *J* = 8.9 Hz, 1H), 8.54 (d, *J* = 9.1 Hz, 1H), 7.83 (d, *J* = 2.2 Hz, 1H), 7.68 (dd, *J* = 9.0, 2.3 Hz, 1H), 7.61 (d, *J* = 2.3 Hz, 1H), 7.40 (d, *J* = 2.8 Hz, 1H), 7.33 (dd, *J* = 8.7, 2.2 Hz, 1H), 7.15 (dd, *J* = 9.1, 2.8 Hz, 1H), 6.61 (d, *J* = 8.7 Hz, 1H), 5.94 (d, *J* = 9.1 Hz, 1H), 5.72 (s, 2H), 4.18 (m, *J* = 23.4, 12.0, 6.1 Hz, 1H), 3.86 (d, *J* = 6.6 Hz, 2H), 2.17 (m, *J* = 13.4, 6.7 Hz, 1H), 1.62 (m, *J* = 14.4, 5.8 Hz, 2H), 1.56 – 1.34 (m, 6H), 1.09 (d, *J* = 6.7 Hz, 6H), 0.97 (t, *J* = 7.3 Hz, 6H) ppm. ^{13}C NMR (151 MHz, $CDCl_3$) δ 167.6, 167.5, 166.7, 155.2, 148.5, 138.5, 135.6, 135.4, 133.5, 130.6, 129.2, 123.7, 123.6, 123.1, 122.3, 120.3, 119.1, 118.1, 116.1, 115.2, 112.4, 108.2, 77.4, 77.2, 77.0, 75.1, 49.9, 37.6, 29.9, 28.4, 19.5, 14.2 ppm. HRMS (ESI) m/z calculated for $C_{32}H_{38}Br_2N_4O_4$: $[M+H]^+$ 700.1260 found 701.1484.

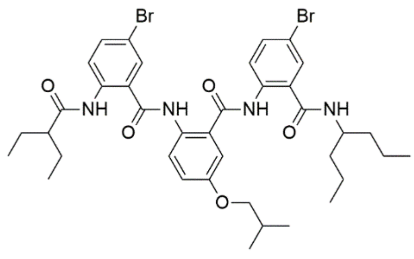

5-bromo-2-(2-(5-bromo-2-(2-ethylbutanamido)benzamido)-5-isobutoxybenzamido)-N-(heptan-4-yl)benzamide (Baa-Box-Baa) (Scheme S2). 13 (50 mg, 0.0714 mmol) was transferred to a dry, Ar purged 25 mL round bottom flask, with a stir bar. This was dissolved in 5 mL of THF. The reaction mixture was cooled to -78°C and was stirred for 5 minutes. Then, (12 μL, 0.086 mmol) of 2-ethylbutyryl chloride was added dropwise followed by pyridine (30 μL, 0.36 mmol) and the stirred for 3 h. Upon completion of the reaction, the mixture was quenched with 50 mL of 5% HCl. The mixture was extracted with DCM (3×25mL). The organic layer was dried Na_2SO_4 and condensed. The product was purified using flash chromatography (stationary phase: silica gel; eluent gradient: 0:1 (v:v) to 1:9 (v:v) of ethyl acetate and hexanes. The product was condensed to afford 48 mg (85%) of **Baa-Box-Baa** as a white solid. ^{1}H NMR (600 MHz, Chloroform-*d*) δ 12.36 (s, 1H), 11.98 (s, 1H), 11.17 (s, 1H), 8.66 (dd, *J* = 17.5, 9.0 Hz, 2H), 8.50 (d, *J* = 9.1 Hz, 1H), 7.97 (d, *J* = 2.3 Hz, 1H), 7.64 (dd, *J* = 8.9, 2.3 Hz, 1H), 7.61 (d, *J* = 2.3 Hz, 1H), 7.57 (dd, *J* = 9.0, 2.2 Hz, 1H), 7.41 (d, *J* = 2.8 Hz, 1H), 7.15 (dd, *J* = 9.1, 2.8 Hz, 1H), 6.12 (d, *J* = 9.1 Hz, 1H), 4.16 (m, *J* = 8.8, 5.0, 4.4 Hz, 1H), 3.84 (d, *J* = 6.6 Hz, 2H), 2.15 (m, *J* = 12.6, 7.9, 4.6 Hz, 2H), 1.74 – 1.66 (m, 2H), 1.64 – 1.52 (m, 4H), 1.49 (m, *J* = 13.7, 9.0, 5.0 Hz, 2H), 1.39 (m, *J* = 16.5, 13.5, 7.2 Hz, 4H), 1.06 (d, *J* = 6.7 Hz, 6H), 0.94 (t, *J* = 7.4 Hz, 12H) ppm. ^{13}C NMR (151 MHz, $CDCl_3$) δ 175.4, 167.6, 167.5, 166.2, 155.7, 139.4, 138.4 135.6, 135.6, 132.9, 130.2, 129.3, 123.7, 123.5, 123.2, 123.0, 122.8, 122.5, 120.3, 116.3, 115.5, 115.2, 112.4, 77.4, 77.2, 77.0, 75.1, 53.0, 49.9, 37.5, 28.4, 25.8, 25.0, 19.5, 19.4, 14.2, 12.2, 12.0 ppm. HRMS (ESI) m/z calculated for $C_{27}H_{35}Br_2N_3O_3$: $[M-H]^-$ 798.2000 found 799.1915.

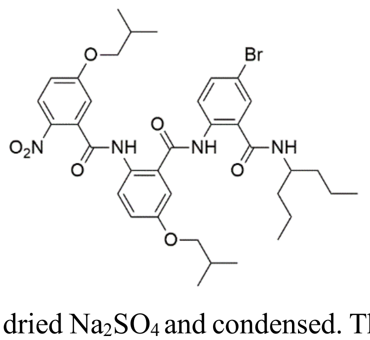

5-bromo-N-(heptan-4-yl)-2-(5-isobutoxy-2-(5-isobutoxy-2-nitrobenzamido)benzamido)benzamide (14) (Scheme S2). 3 (95 mg, 0.40 mmol), HOAt (61 mg, 0.45 mmol), DIC (62 μL, 0.40 mmol), DMAP (1 mg, 0.001 mmol) and pyridine (60 μL, 0.70 mmol) was dissolved in 10 mL of DMF in a dry, argon purged 100 mL round bottom flask with a stir bar. This was stirred for 15 minutes and then **(11)** (50 mg, 0.099 mmol) in 5 mL of DCM, was transferred and stirred overnight. The progress of the reaction was monitored via TLC. The mixture quenched with 100 mL of 5% HCl then was extracted with DCM (3×25mL). The organic layer was dried Na_2SO_4 and condensed. The product was purified using flash chromatography (stationary phase: silica gel; eluent gradient: 0:1 (v:v) to 2:4 (v:v) of ethyl acetate and hexanes. This afforded 68 mg (95%) of **(14)** as a dark yellow solid. ^{1}H NMR (600 MHz, Chloroform-*d*) δ 12.16 (s, 1H), 11.26 (s, 1H), 8.65 (d, *J* = 9.1

Hz, 1H), 8.50 – 8.46 (m, 1H), 8.14 (d, J = 9.1 Hz, 1H), 7.59 – 7.54 (m, 2H), 7.37 (d, J = 2.8 Hz, 1H), 7.16 (dd, J = 9.1, 2.8 Hz, 1H), 7.08 (d, J = 2.7 Hz, 1H), 7.00 (dd, J = 9.1, 2.7 Hz, 1H), 5.87 (d, J = 9.1 Hz, 1H), 4.20 – 4.10 (m, 1H), 3.83 (d, J = 6.5 Hz, 4H), 2.14 (m, J = 13.3, 10.1, 6.7 Hz, 2H), 1.64 – 1.53 (m, 2H), 1.53 – 1.44 (m, 2H), 1.39 (m, J = 20.8, 9.7, 6.5 Hz, 4H), 1.05 (dd, J = 15.5, 6.7 Hz, 12H), 0.95 (t, J = 7.3 Hz, 6H). ppm. ^{13}C NMR (151 MHz, $CDCl_3$) δ 167.4, 164.6, 163.7, 155.6, 138.9, 138.4, 136.2, 135.3, 133.3, 129.4, 127.5, 123.7, 123.4, 123.3, 121.9, 120.4, 116.1, 115.6, 115.2, 114.3, 112.4, 77.4, 77.2, 77.0, 75.5, 75.1, 49.9, 37.5, 28.4, 28.3, 19.5, 19.4, 19.3, 14.2 ppm. HRMS (ESI) m/z calculated for $C_{36}H_{45}BrN_4O_7$: $[M+Na]^+$ 724.2472, found 725.2702.

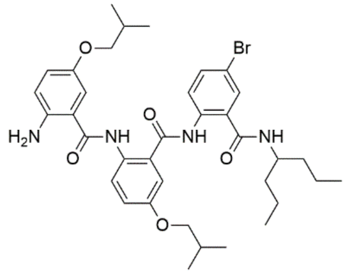

2-amino-N-(2-((4-bromo-2-(heptan-4-ylcarbamoyl)phenyl)carbamoyl)-4-isobutoxyphenyl)-5-isobutoxybenzamide (15) (Scheme S2). **14** (274 mg, 0.379 mmol) and $Co_2(CO)_8$ (259 mg, 0.76 mmol) were placed in a 25-mL pressure tube with a magnetic stir bar in it. While purging with argon, 10 mL of 1,2-dimethoxyethane (DME) and five drops of DI water was added to the tube and tightly closed. While mixing, the pressure tube was immersed in a temperature-controlled oil bath. The mixture was heated to 90 °C and stirred for four hours. It was taken out of the oil bath and allowed to cool to room temperature prior to opening it. The reaction mixture was filtered; the filtrate was collected and condensed then diluted with 20 mL DCM, and washed with water (100 mL). The organic layer was collected, dried over Na_2SO_4, and concentrated in vacuo. The product was purified using flash chromatography (stationary phase: silica gel; eluent gradient: from 0:1 (v:v) to 1:5 ratio of ethyl acetate and hexanes) to afford 123 mg (47%) of **(15)** as a yellow solid. ^{1}H NMR (600 MHz, Chloroform-*d*) δ 12.21 (s, 1H), 11.72 (s, 1H), 8.67 (d, J = 8.9 Hz, 1H), 8.63 (d, J = 9.1 Hz, 1H), 7.61 – 7.58 (m, 1H), 7.58 – 7.55 (m, 1H), 7.37 (d, J = 2.8 Hz, 1H), 7.27 (d, J = 2.8 Hz, 1H), 7.13 (dd, J = 9.1, 2.8 Hz, 1H), 6.92 (dd, J = 8.8, 2.7 Hz, 1H), 6.66 (d, J = 8.8 Hz, 1H), 5.93 (d, J = 9.1 Hz, 1H), 5.40 – 5.37 (m, 2H), 4.16 (m, J = 8.7, 4.9, 4.3 Hz, 1H), 3.83 (d, J = 6.6 Hz, 2H), 3.78 (d, J = 6.6 Hz, 2H), 2.18 – 2.06 (m, 2H), 1.58 (m, J = 11.4, 9.6, 5.8 Hz, 2H), 1.51 – 1.33 (m, 6H), 1.06 (d, J = 6.7 Hz, 12H), 0.94 (t, J = 7.3 Hz, 6H) ppm. ^{13}C NMR (151 MHz, $CDCl_3$) δ 167.8, 167.6, 167.5, 155.0, 151.0, 143.8, 138.7, 135.3, 134.1, 129.3, 123.4, 123.1, 122.0, 121.8, 120.4, 119.2, 116.7, 115.9, 112.3, 112.1, 77.4, 77.2, 77.0, 75.7, 75.1, 49.9, 37.6, 28.5, 28.4, 19.6, 19.5, 14.2 ppm. HRMS (ESI) m/z calculated for $C_{36}H_{47}BrN_4O_5$: $[M+H]^+$ 694.2709 found 697.2772.

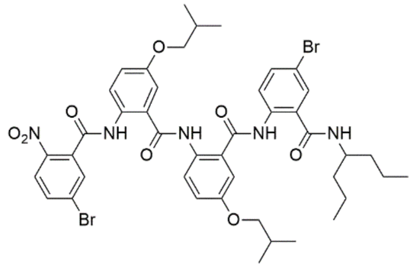

5-bromo-N-(2-((2-((4-bromo-2-(heptan-4-ylcarbamoyl)phenyl)carbamoyl)-4-isobutoxyphenyl)carbamoyl)-4-isobutoxyphenyl)-2-nitrobenzamide (16) (Scheme S2). **5** (172 mg, 0.70 mmol), HOAt (108 mg, 0.79 mmol), DIC (109 µL, 0.70 mmol), DMAP (3 mg, 0.02 mmol) and pyridine (100 µL, 1.23 mmol) was dissolved in 15 mL of DMF in a dry, argon purged 100 mL round bottom flask with a stir bar. This was stirred for 15 minutes and then **(15)** (122 mg, 0.176 mmol) in 5 mL of DCM, was transferred and stirred overnight. The progress of the reaction was monitored via TLC. The mixture quenched with 100 mL of 5% HCl then was extracted with DCM (3×25mL). The organic layer was dried Na_2SO_4 and condensed. The product was purified using flash chromatography (stationary phase: silica gel; eluent gradient: 0:1 (v:v) to 1:4 (v:v) of ethyl acetate and hexanes. This afforded 91 mg (56%) of **(16)** as a yellow solid. ^{1}H NMR (600 MHz, Chloroform-*d*) δ 12.36 (s, 1H), 12.10 (s, 1H), 11.60 (s, 1H), 8.69 – 8.65 (m, 1H), 8.63 (d, J = 9.1 Hz, 1H), 8.47 (d, J = 9.1 Hz, 1H), 7.96 (d, J = 8.7 Hz, 1H), 7.82 (d, J = 2.1 Hz, 1H), 7.72 (dd, J = 8.7, 2.1 Hz, 1H), 7.62 – 7.56 (m, 2H), 7.45 (d, J = 2.8 Hz, 1H), 7.40 (d, J = 2.8 Hz, 1H), 7.13 (ddd, J = 20.8, 9.1, 2.8 Hz, 2H), 5.93 (d, J = 9.1 Hz, 1H), 4.16 (m, J = 8.6, 3.6 Hz, 1H), 3.86 (d, J = 6.6 Hz, 2H), 3.83 (d, J = 6.6 Hz, 2H), 2.15 (m, J = 19.9, 6.7 Hz, 2H), 1.63 – 1.55 (m, 2H), 1.53 – 1.31 (m, 6H), 1.07 (dd, J =

21.5, 6.7 Hz, 12H), 0.94 (t, *J* = 7.3 Hz, 6H) ppm. ^{13}C NMR (151 MHz, $CDCl_3$) δ 167.6, 167.5, 167.0, 162.7, 155.7, 145.6, 138.6, 135.5, 135.2, 133.8, 133.1, 133.0, 131.9, 129.3, 129.0, 126.4, 123.6, 123.4, 123.0, 122.3, 120.4, 120.1, 116.2, 115.2, 112.5, 77.4, 77.2, 77.0, 75.2, 75.1, 49.9, 37.6, 28.5, 28.4, 19.5, 19.5, 19.4, 14.2. HRMS (ESI) m/z calculated for $C_{43}H_{49}Br_2N_5O_8$: $[M+Na]^+$ 921.1911, found 946.1795.

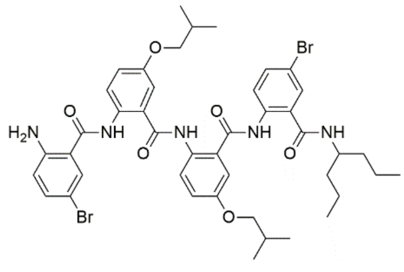

2-amino-5-bromo-N-(2-((2-((4-bromo-2-(heptan-4-ylcarbamoyl)phenyl)carbamoyl)-4-isobutoxyphenyl)carbamoyl)-4-isobutoxyphenyl)benzamide (17) (Scheme S2). (16) (48 mg, 0.0521 mmol) and $Co_2(CO)_8$ (36 mg, 0.10 mmol) were placed in a 25-mL pressure tube with a magnetic stir bar in it. While purging with argon, 10 mL of 1,2-dimethoxyethane (DME) and five drops of DI water was added to the tube and tightly closed. While mixing, the pressure tube was immersed in a temperature-controlled oil bath. The mixture was heated to 90 °C and stirred for four hours. It was taken out of the oil bath and allowed to cool to room temperature prior to opening it. The reaction mixture was filtered; the filtrate was collected and condensed then diluted with 20 mL DCM, and washed with water (100 mL). The organic layer was collected, dried over Na_2SO_4, and concentrated in vacuo. The product was purified using flash chromatography (stationary phase: silica gel; eluent gradient: from 0:1 (v:v) to 1:4 ratio of ethyl acetate and hexanes) to afford crude of **(17)** as a yellow solid. HRMS (ESI) m/z calculated for $C_{43}H_{51}Br_2N_5O_6$: $[M-H]^-$ 891.2197 found 892.2111.

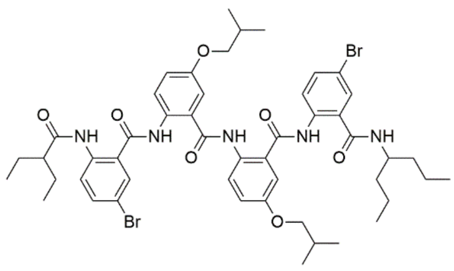

5-bromo-2-(2-(2-(5-bromo-2-(2-ethylbutanamido)benzamido)-5-isobutoxybenzamido)-5-isobutoxybenzamido)-N-(heptan-4-yl)benzamide (Baa-Box-Box-Baa) (Scheme S2). The crude mixture of **(17)** (0.0521 mmol) was transferred to a dry, Ar purged 25 mL round bottom flask, with a stir bar. This was dissolved in 5 mL of THF. The reaction mixture was cooled to -78°C and was stirred for 5 minutes. Then, (3 µL, 0.021 mmol) of 2-ethylbutyryl chloride was added dropwise followed by pyridine (7 µL, 0.36 mmol) and the stirred for 3 h. Upon completion of the reaction, the mixture was quenched with 50 mL of 5% HCl. The mixture was extracted with DCM (3×25mL). The organic layer was dried Na_2SO_4 and condensed. The product was purified using flash chromatography (stationary phase: silica gel; eluent gradient: 0:1 (v:v) to 1:9 (v:v) of ethyl acetate and hexanes. The product was condensed to afford 10 mg (19%) of **Baa-Box-Box-Baa** as a white solid. ^1H NMR (600 MHz, Chloroform-*d*) δ 12.38 (s, 1H), 12.21 (s, 1H), 12.08 (s, 1H), 11.20 (s, 1H), 8.71 – 8.65 (m, 3H), 8.51 (d, *J* = 9.1 Hz, 1H), 7.99 (d, *J* = 2.3 Hz, 1H), 7.61 – 7.56 (m, 3H), 7.48 (d, *J* = 2.8 Hz, 1H), 7.42 (d, *J* = 2.8 Hz, 1H), 7.16 (ddd, *J* = 21.7, 9.1, 2.8 Hz, 2H), 5.91 (d, *J* = 9.1 Hz, 1H), 4.17 (m, *J* = 13.2, 4.5, 3.9 Hz, 1H), 3.87 (d, *J* = 6.6 Hz, 2H), 3.84 (d, *J* = 6.6 Hz, 2H), 2.16 (m, *J* = 17.7, 13.2, 6.9 Hz, 2H), 1.75 – 1.67 (m, 2H), 1.65 – 1.53 (m, 4H), 1.52 – 1.33 (m, 6H), 1.08 (dd, *J* = 17.8, 6.7 Hz, 12H), 0.94 (t, *J* = 7.4, 4.1 Hz, 12H) ppm. ^{13}C NMR (151 MHz, $CDCl_3$) δ 175.3, 167.8, 167.5, 167.1, 166.2, 155.7, 139.5, 138.6, 135.6, 135.4, 133.3, 132.9, 130.3, 129.3, 123.7, 123.5, 123.3, 123.1, 122.8, 121.9, 120.6, 120.0, 116.2, 115.5, 112.6, 112.4, 77.4, 77.2, 77.0, 75.2, 75.1, 53.1, 49.9, 37.6, 29.9, 28.5, 28.4, 25.9, 19.6, 19.5, 14.2, 12.2 ppm. HRMS (ESI) m/z calculated for $C_{49}H_{61}Br_2N_5O_7$: $[M-H]^-$ 989.2934 found 990.2853.

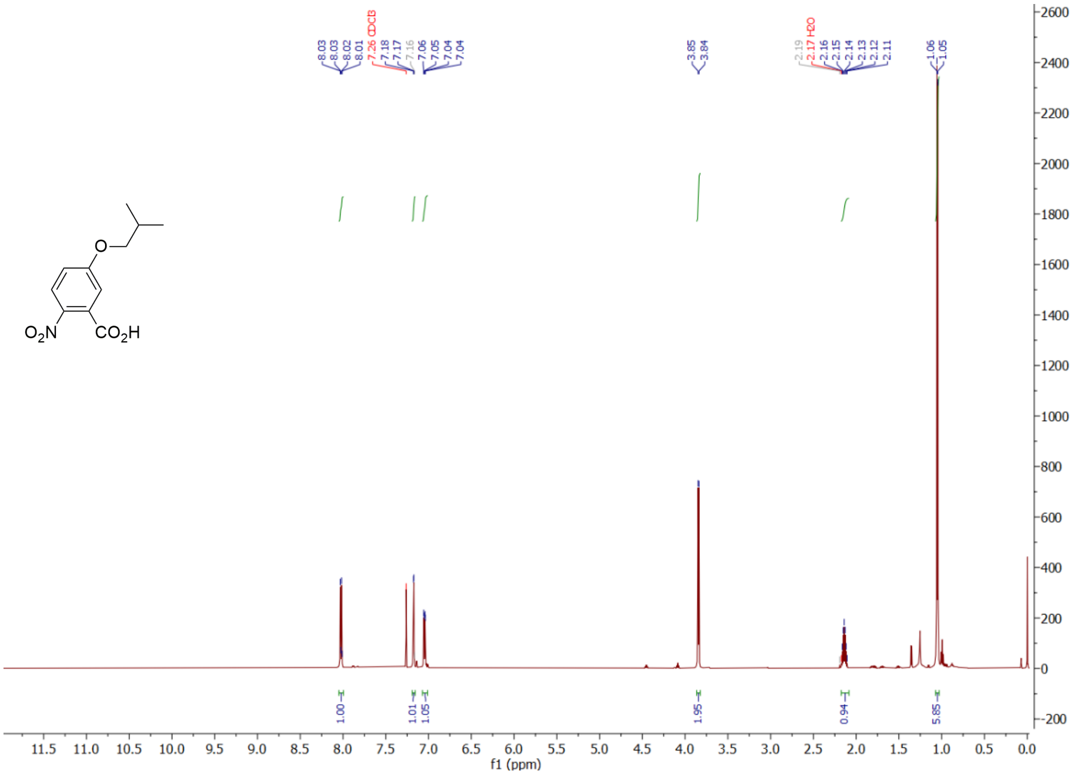

a

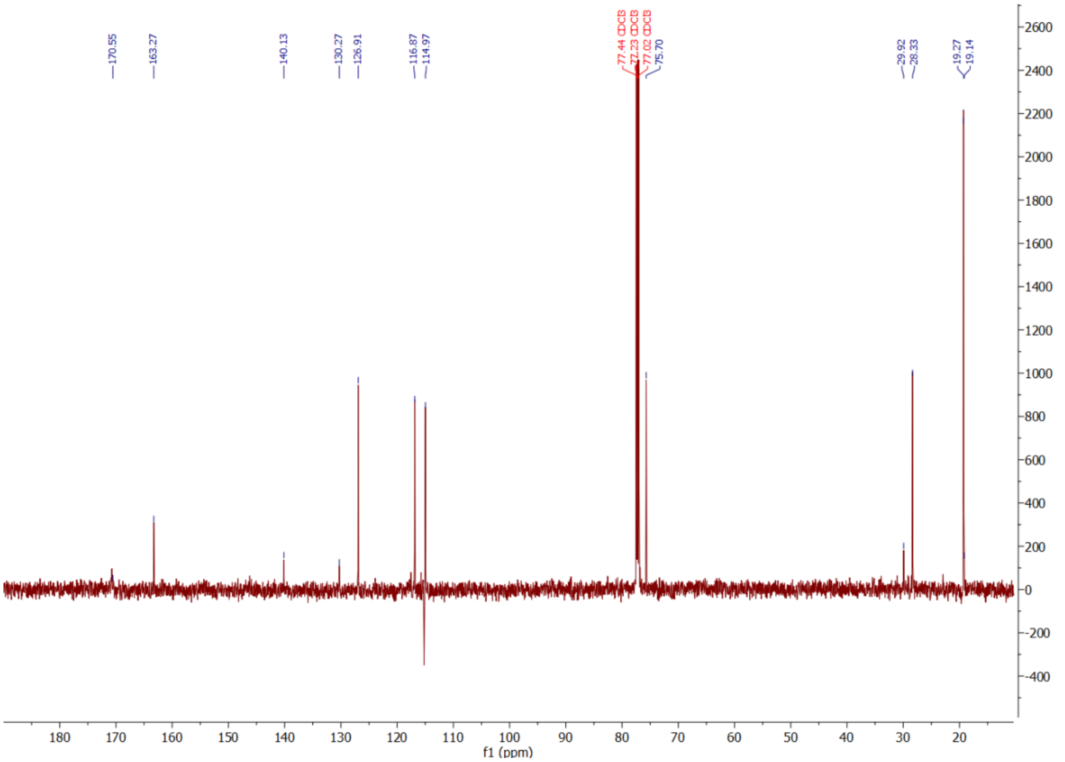

b

Figure S1. (a) ^{1}H NMR of **3** (600 MHz, $CDCl_3$); (b) ^{13}C NMR of **3** (151 MHz, $CDCl_3$).

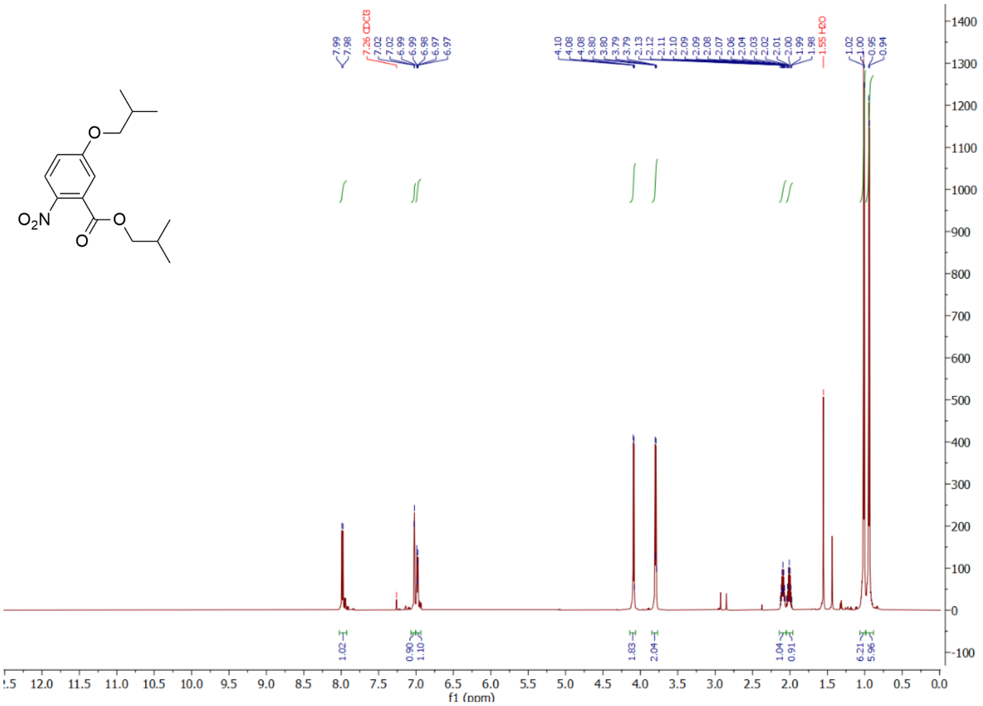

a

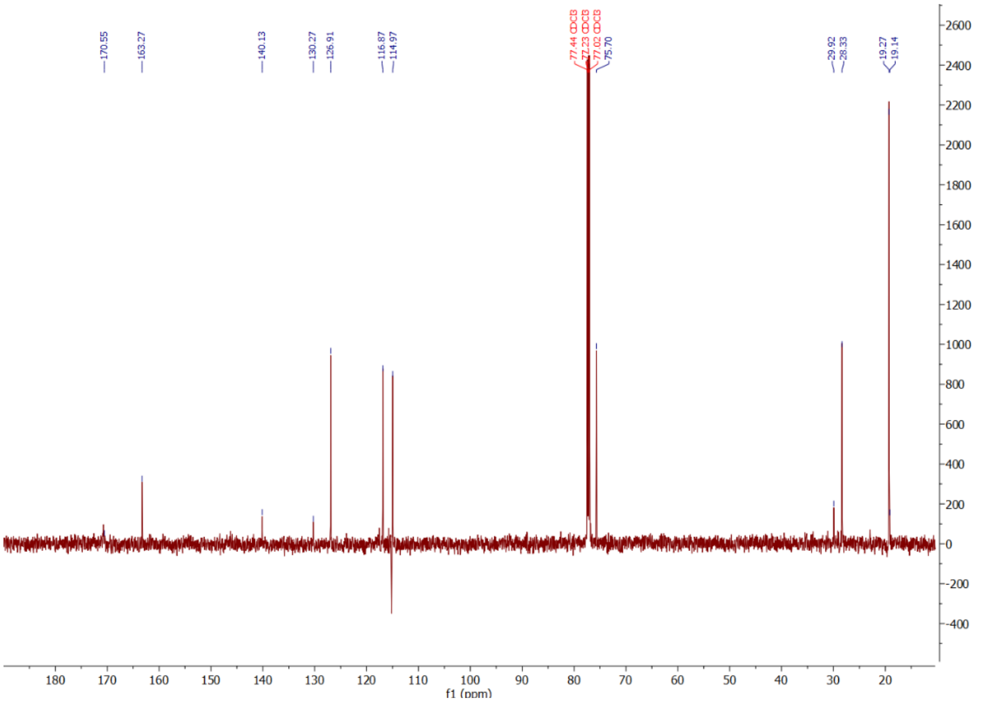

b

Figure S2. (a) ^{1}H NMR of **4** (600 MHz, $CDCl_3$); (b) ^{13}C NMR of **4** (151 MHz, $CDCl_3$).

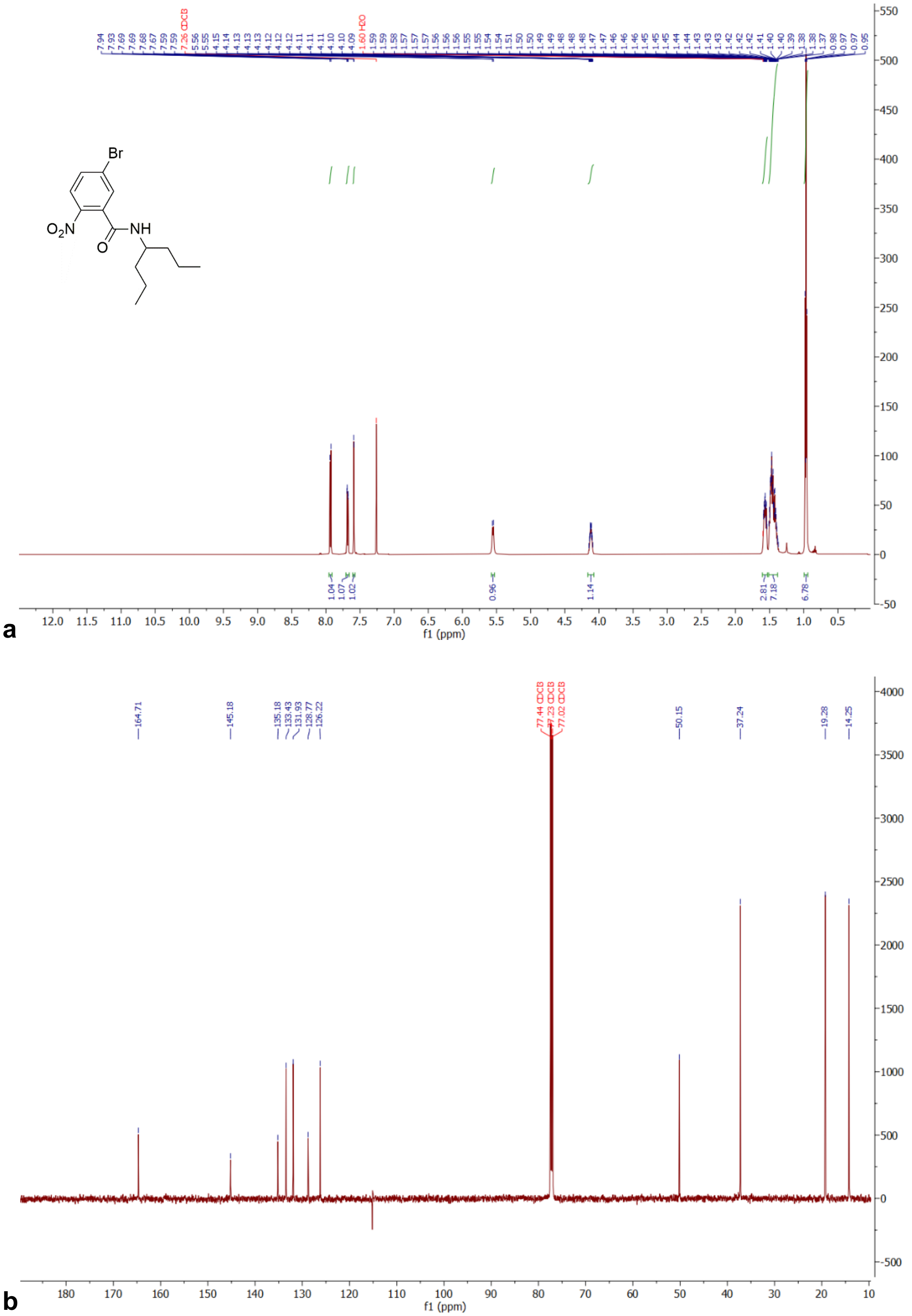

Figure S3. (a) ^{1}H NMR of **6** (600 MHz, $CDCl_3$); (b) ^{13}C NMR of **6** (151 MHz, $CDCl_3$).

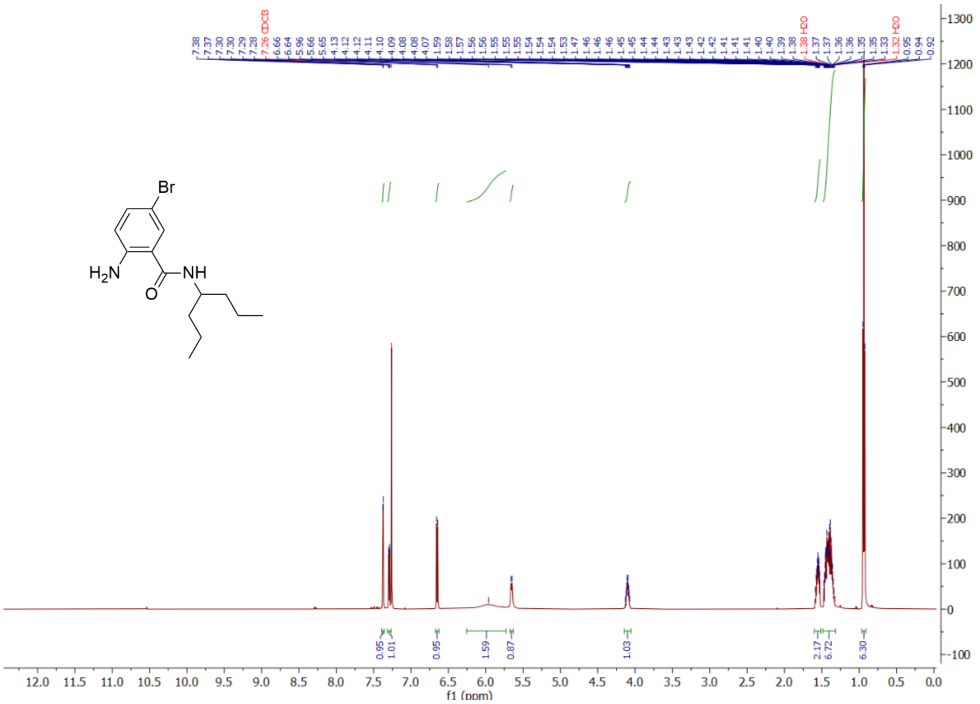

a

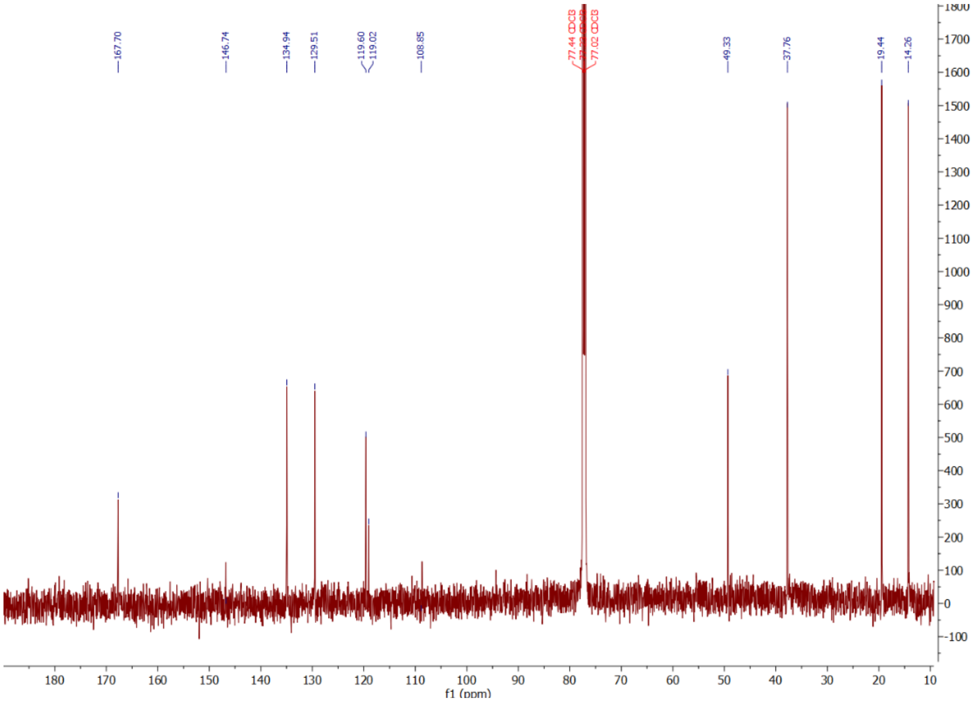

b

Figure S4. (a) ^{1}H NMR of **7** (600 MHz, $CDCl_3$); (b) ^{13}C NMR of **7** (151 MHz, $CDCl_3$).

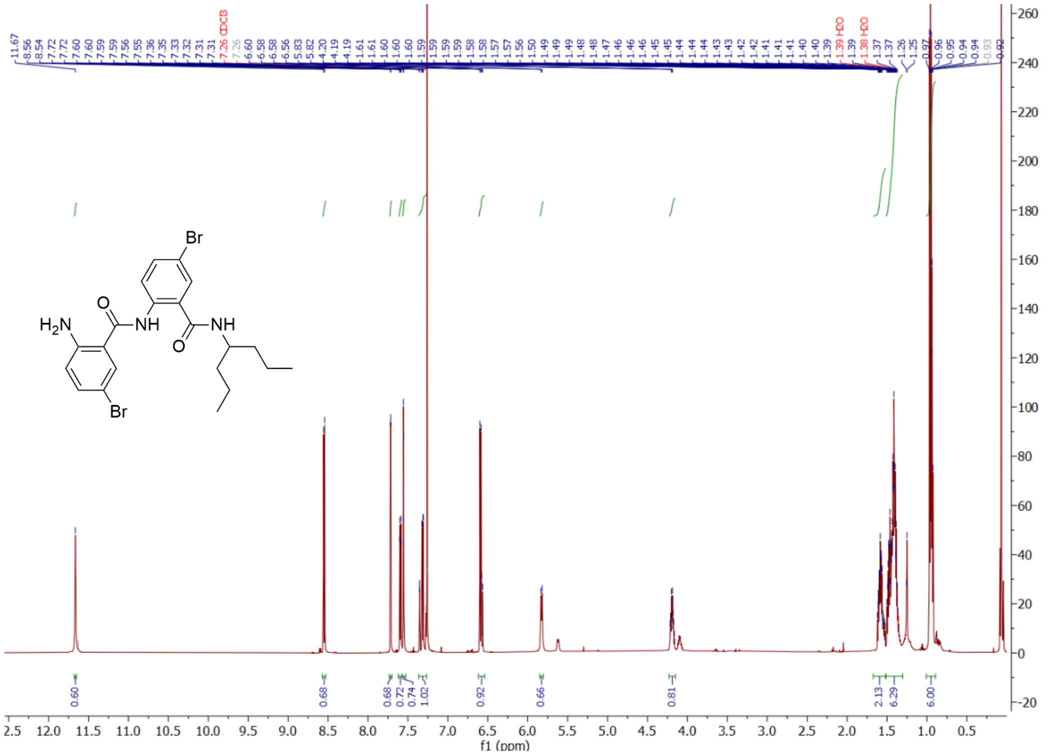

a

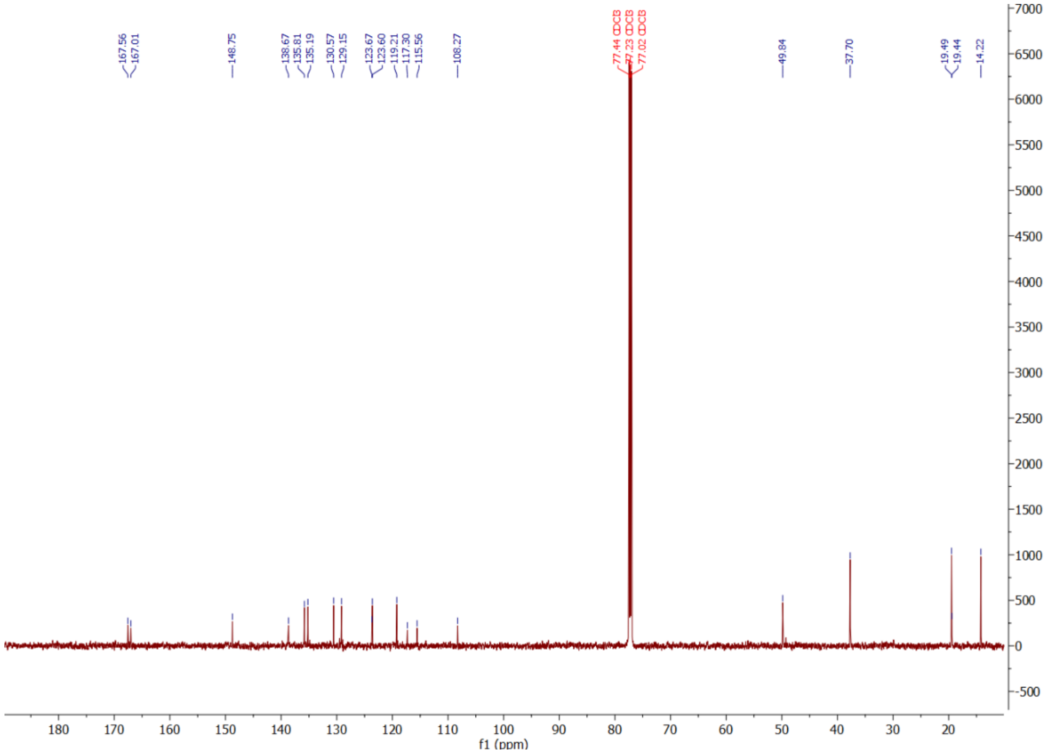

b

Figure S5. (a) ^{1}H NMR of **9** (600 MHz, $CDCl_3$); (b) ^{13}C NMR of **9** (151 MHz, $CDCl_3$).

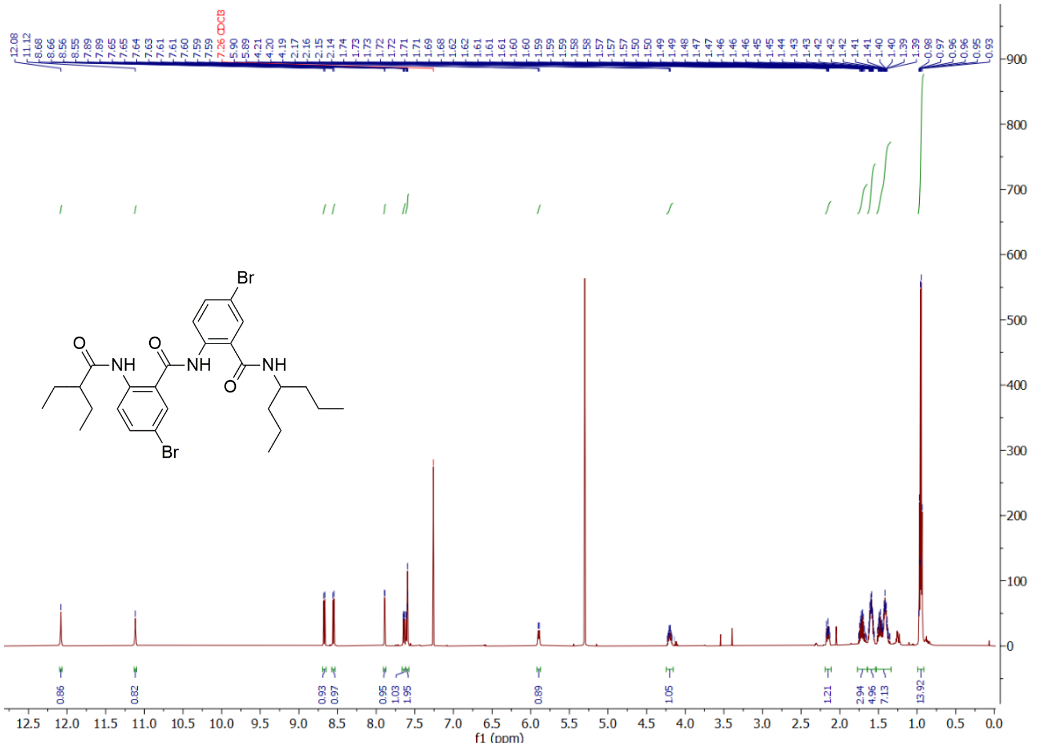

a

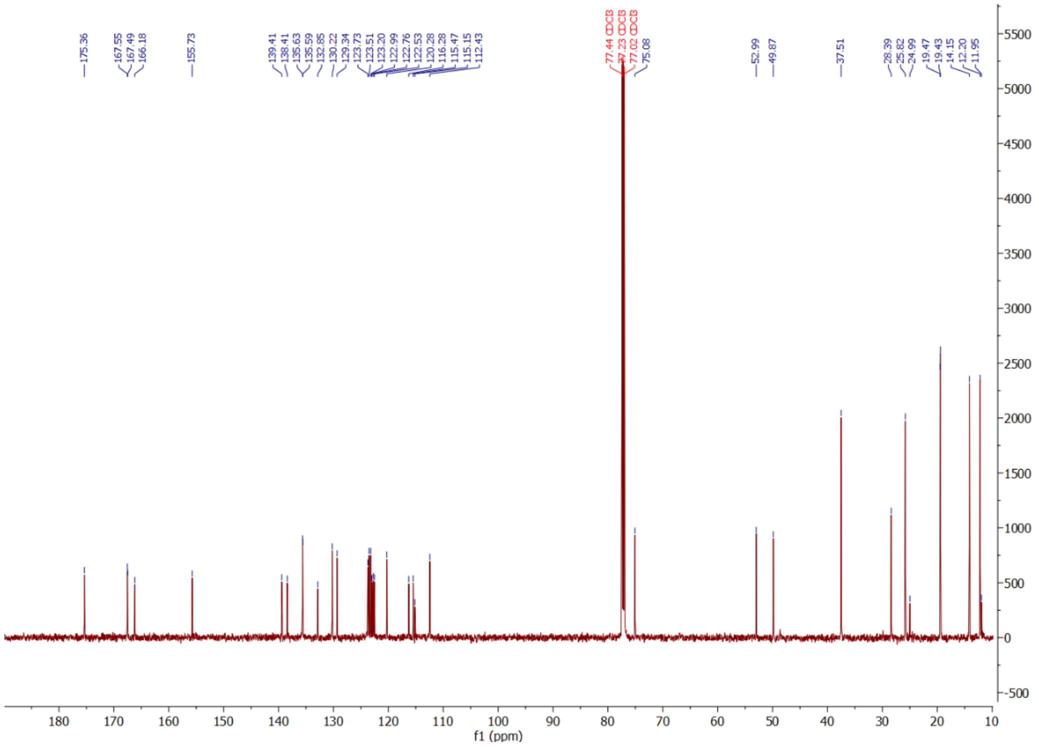

b

Figure S6. (a) ^{1}H NMR of **Baa-Baa** (600 MHz, $CDCl_3$); (b) ^{13}C NMR of **Baa-Baa** (151 MHz, $CDCl_3$).

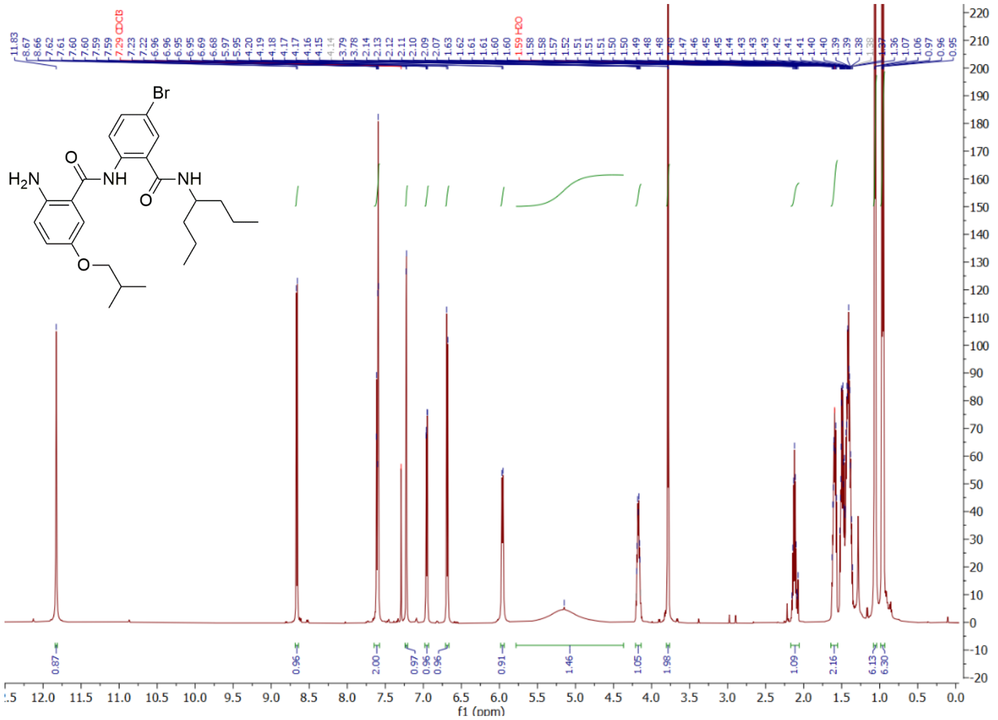

a

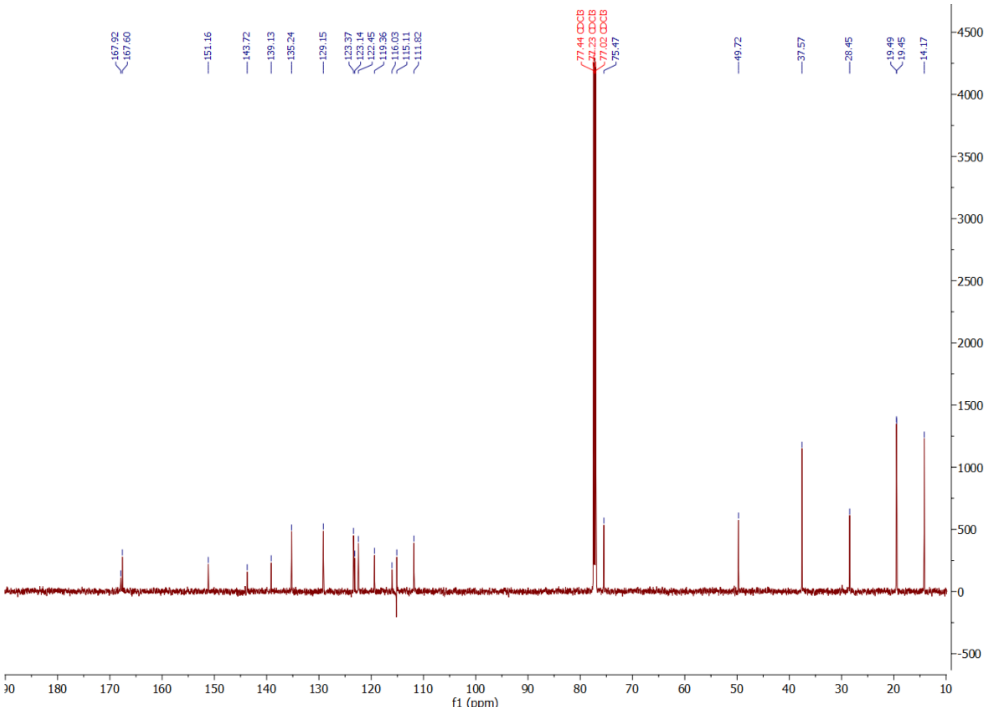

b

Figure S7. (a) ^{1}H NMR of **11** (600 MHz, $CDCl_3$); (b) ^{13}C NMR of **11** (151 MHz, $CDCl_3$).

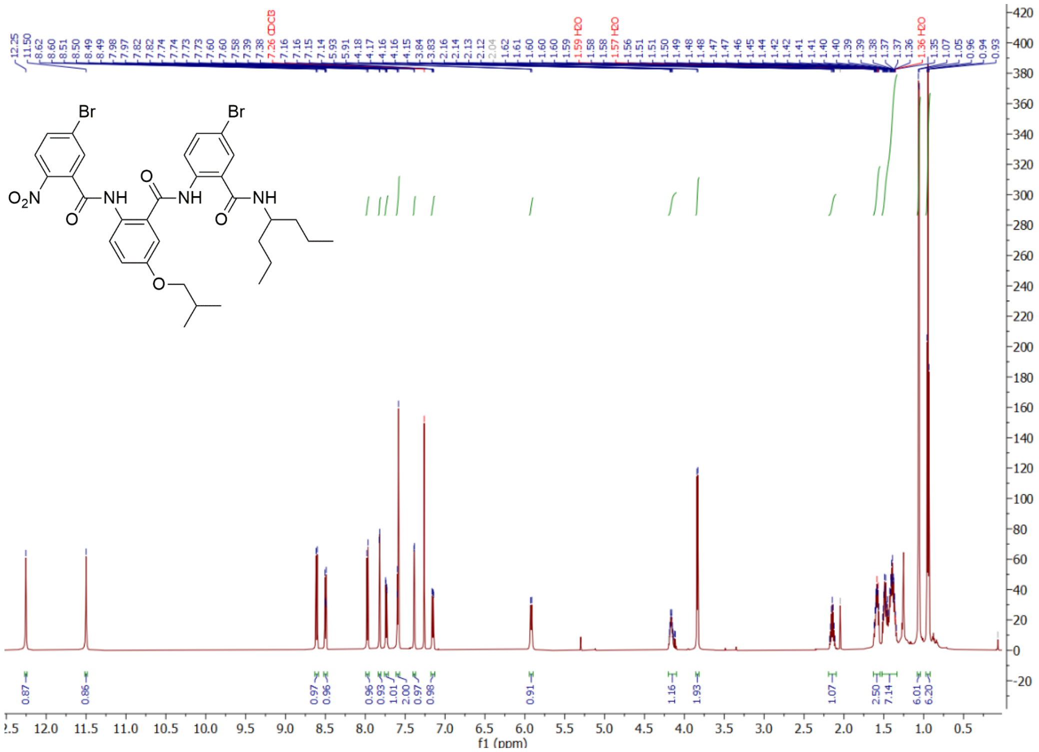

a

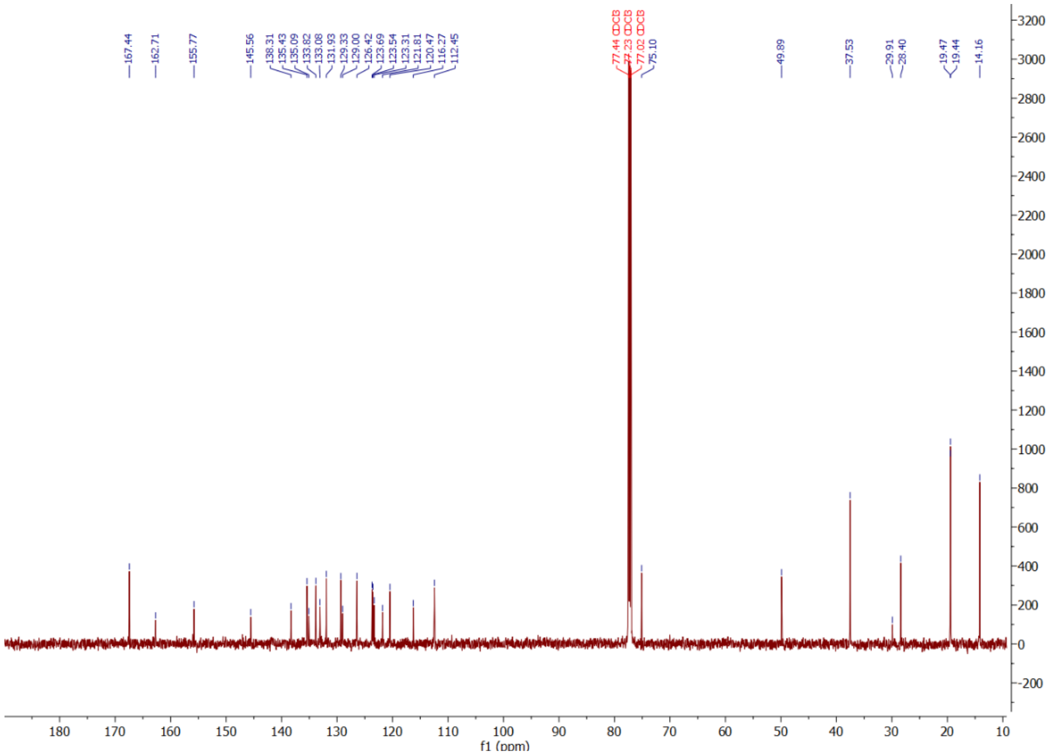

b

Figure S8. (a) ^{1}H NMR of **12** (600 MHz, $CDCl_3$); (b) ^{13}C NMR of **12** (151 MHz, $CDCl_3$).

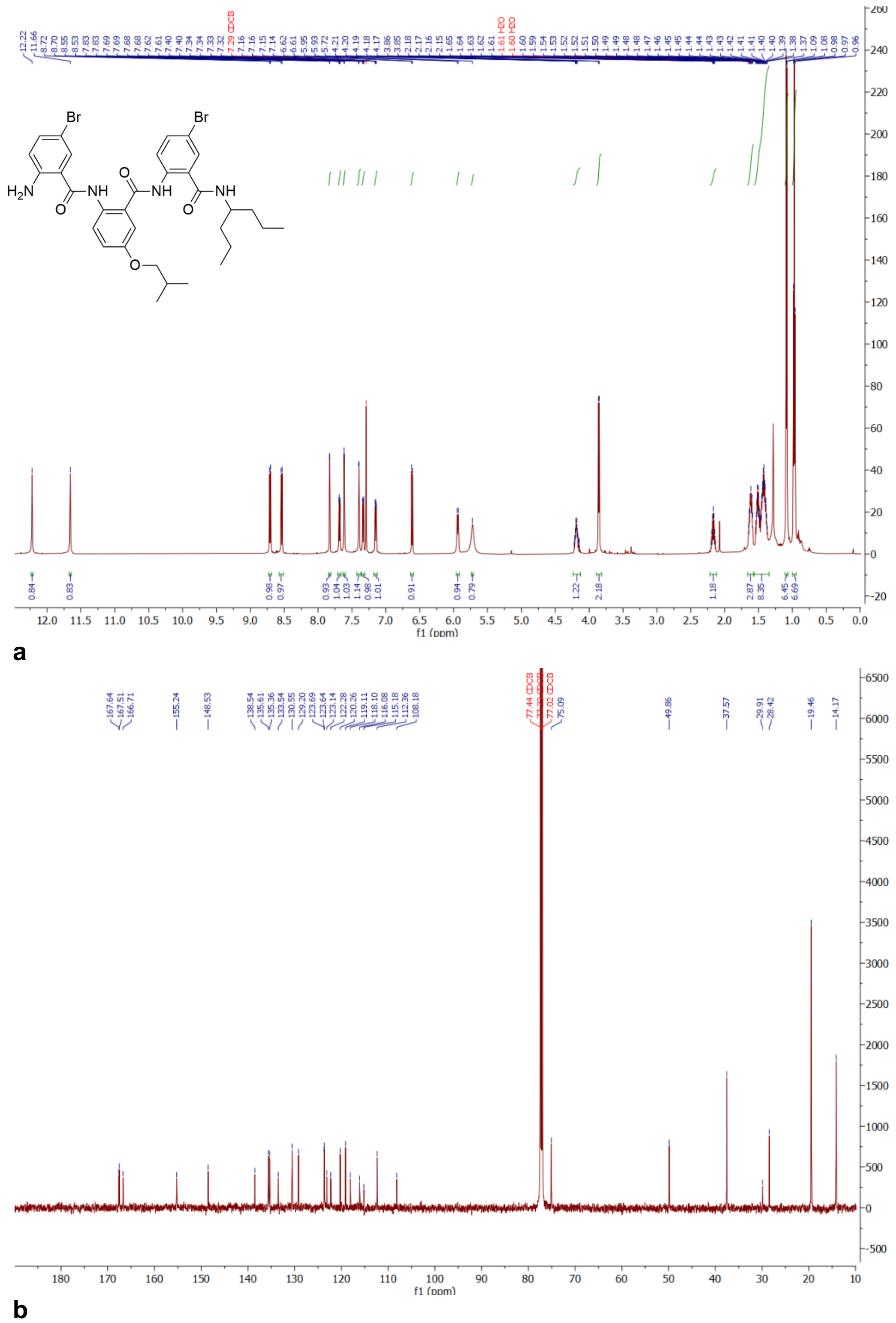

Figure S9. (a) ^{1}H NMR of **13** (600 MHz, $CDCl_3$); (b) ^{13}C NMR of **13** (151 MHz, $CDCl_3$).

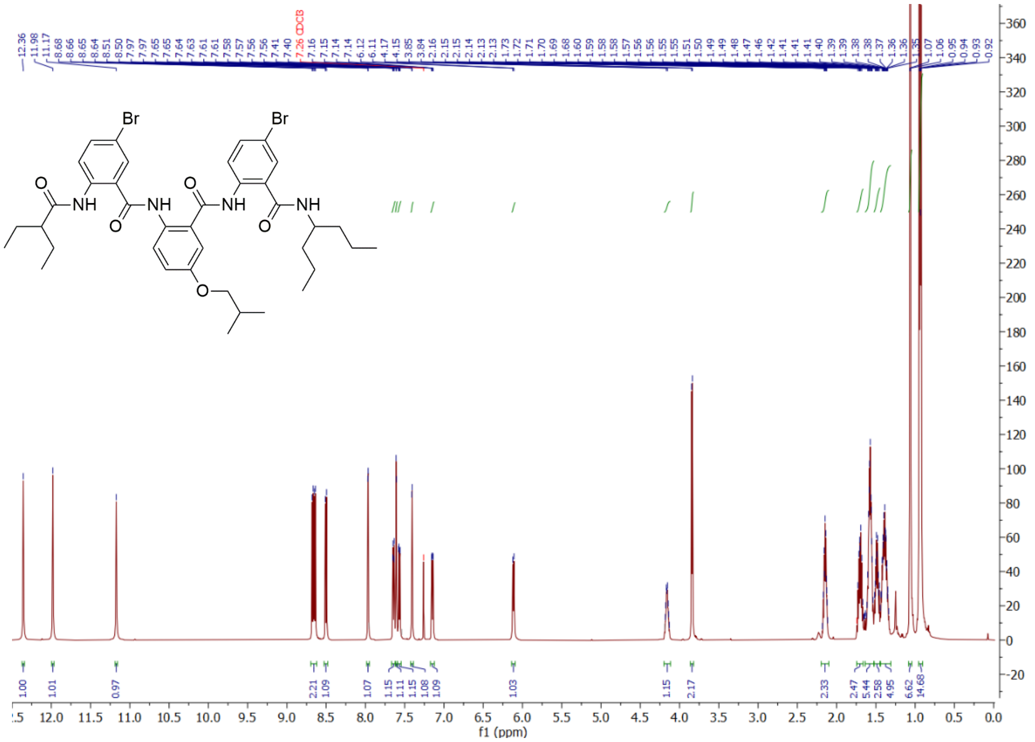

a

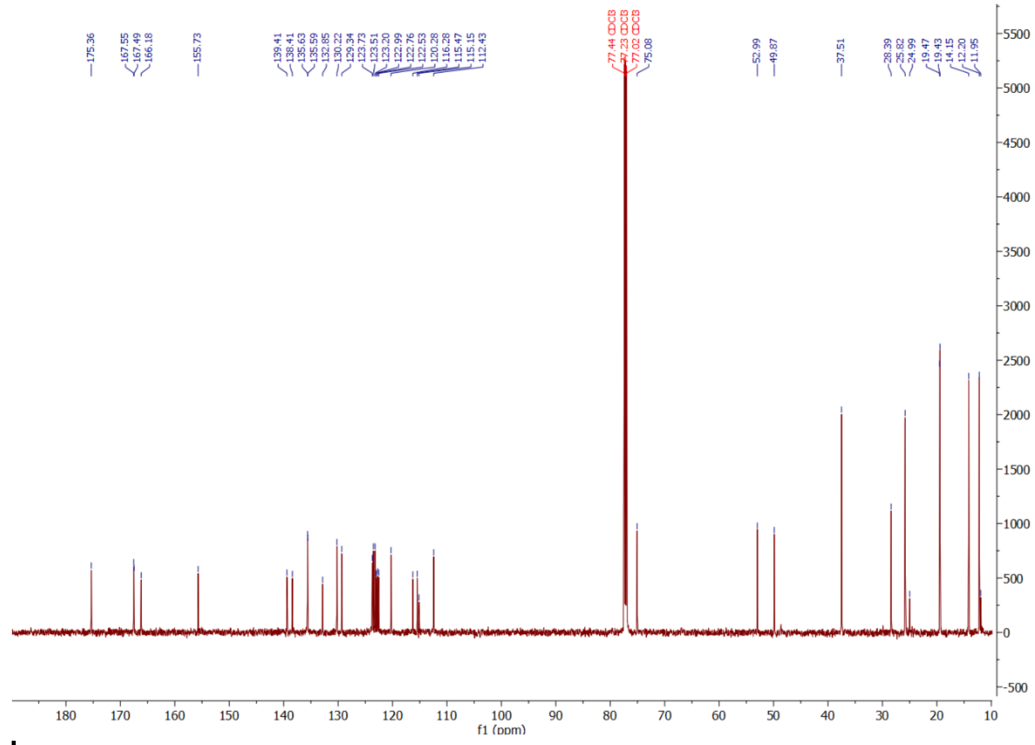

b

Figure S10. (a) ^{1}H NMR of **Baa-Box-Baa** (600 MHz, $CDCl_3$); (b) ^{13}C NMR of **Baa-Box-Baa** (151 MHz, $CDCl_3$).

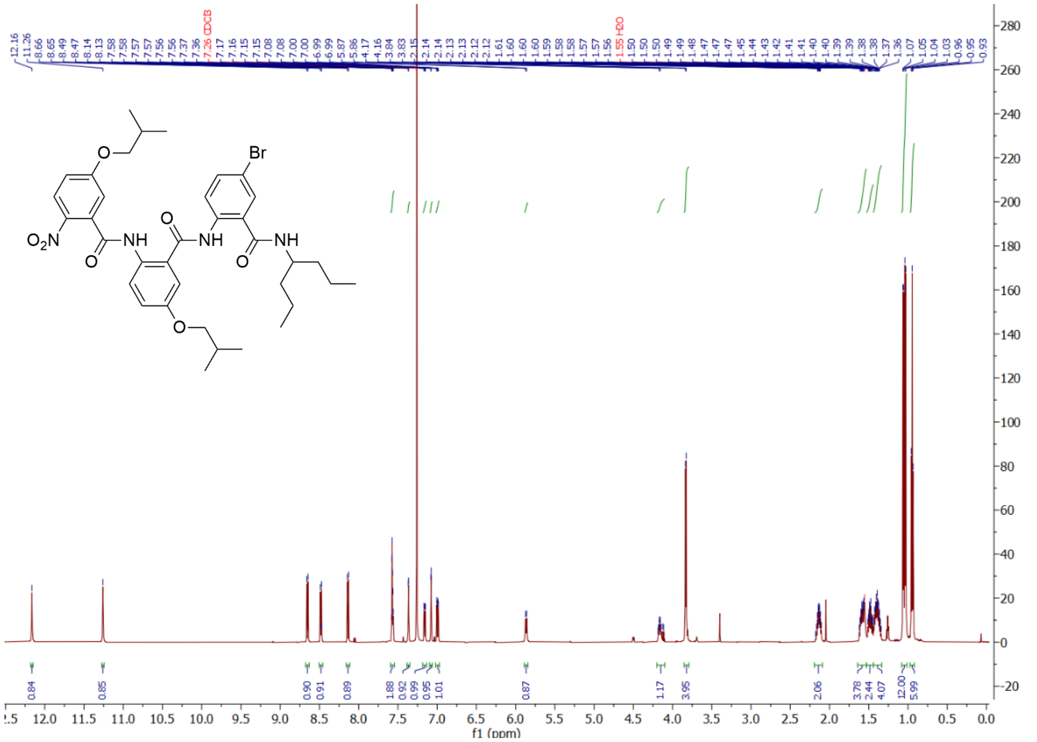

a

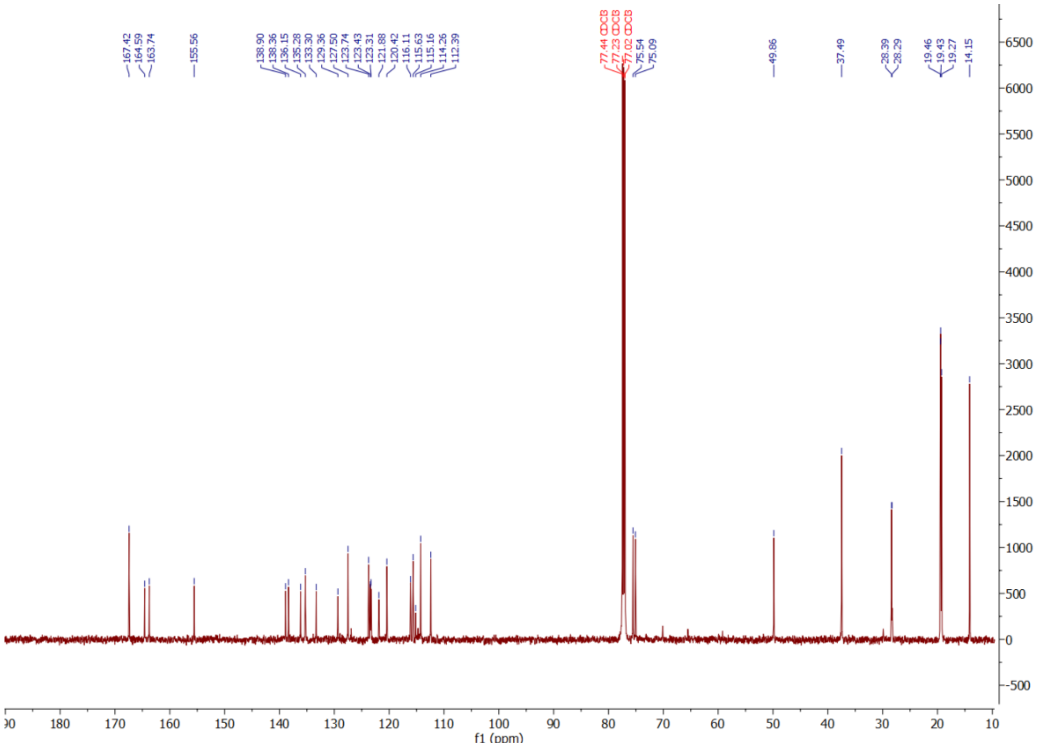

b

Figure S11. (a) ^{1}H NMR of **14** (600 MHz, $CDCl_3$); (b) ^{13}C NMR of **14** (151 MHz, $CDCl_3$).

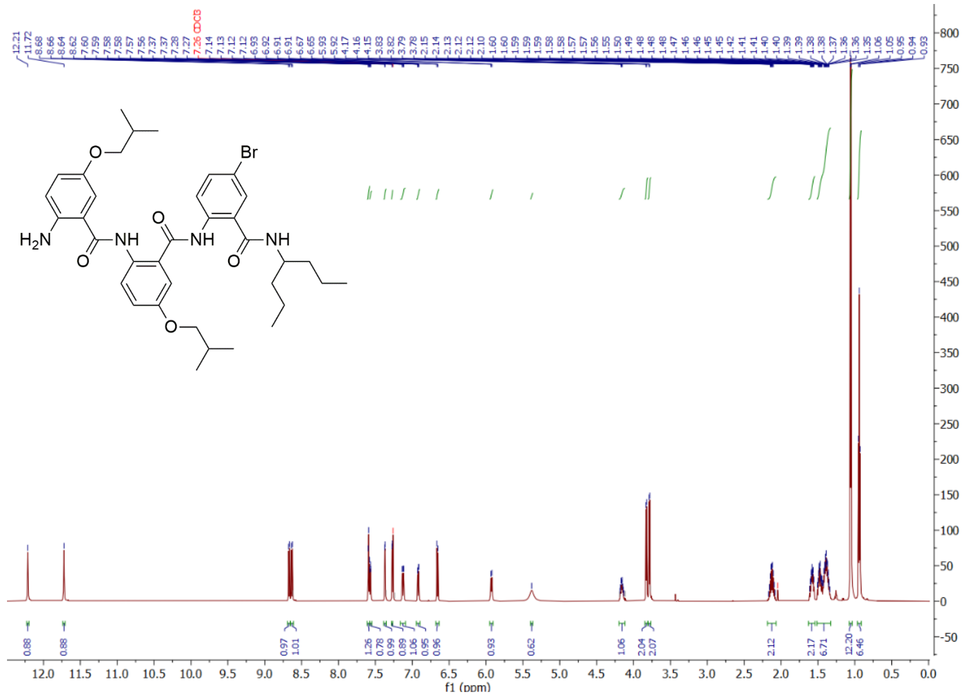

a

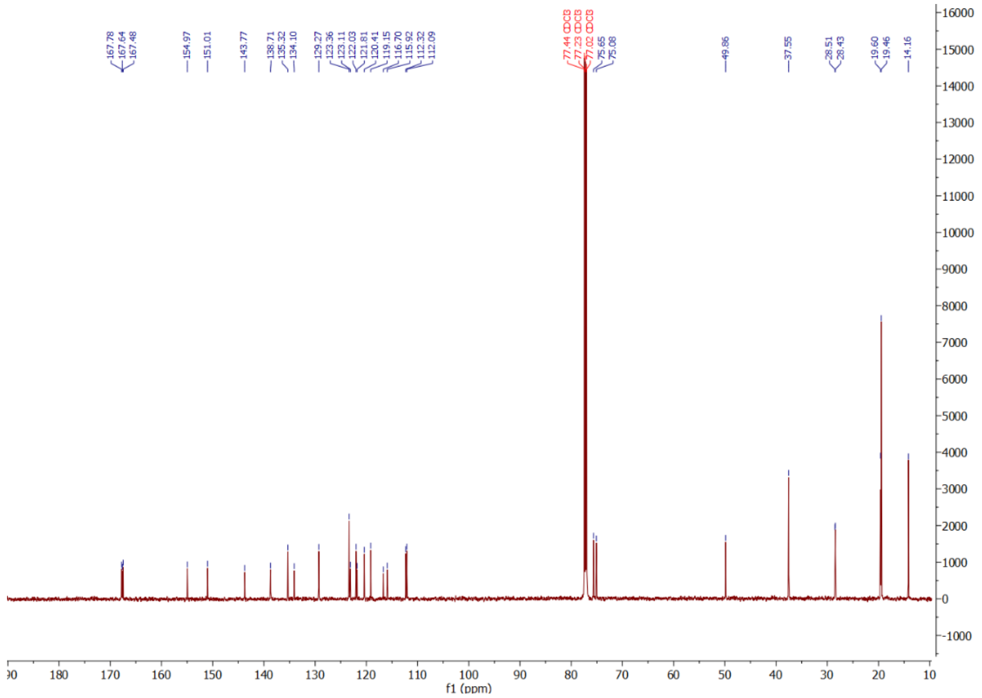

b

Figure S12. (a) ^{1}H NMR of **15** (600 MHz, $CDCl_3$); (b) ^{13}C NMR of **15** (151 MHz, $CDCl_3$).

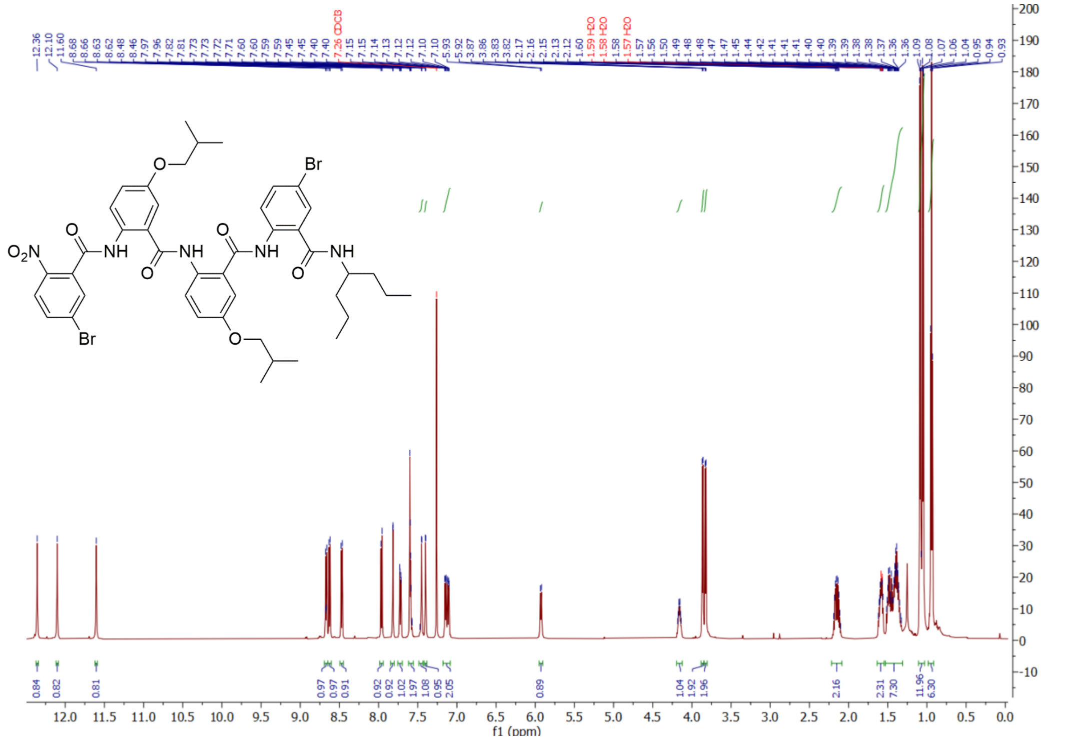

a

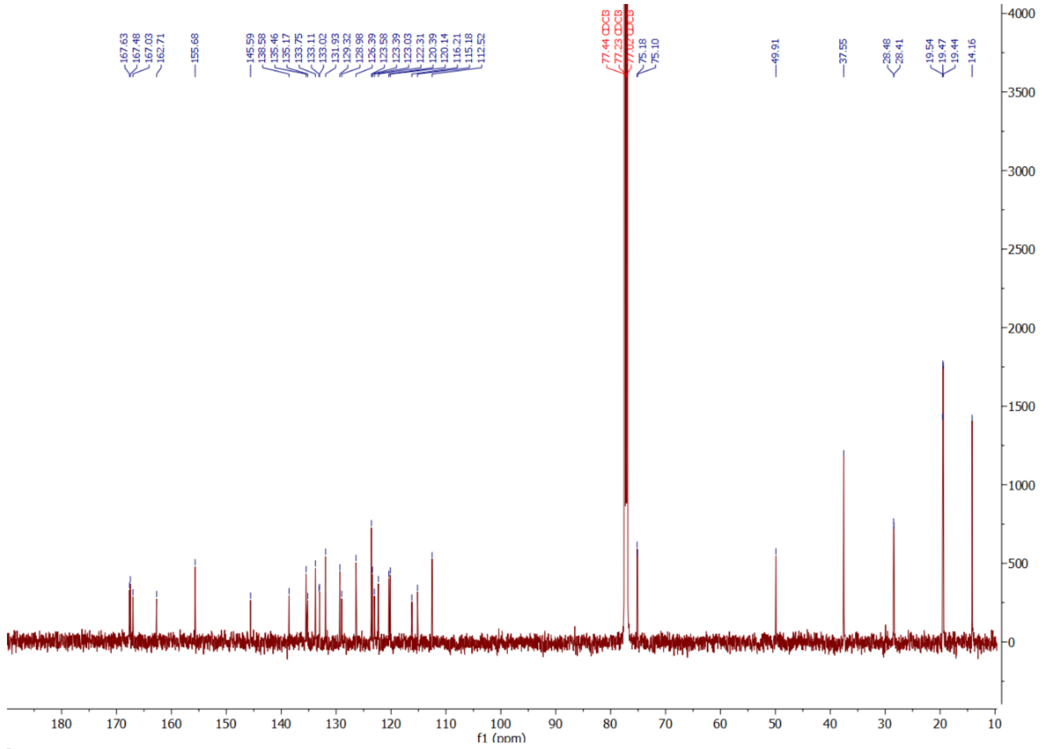

b

Figure S13. (a) ^{1}H NMR of **16** (600 MHz, $CDCl_3$); (b) ^{13}C NMR of **16** (151 MHz, $CDCl_3$).

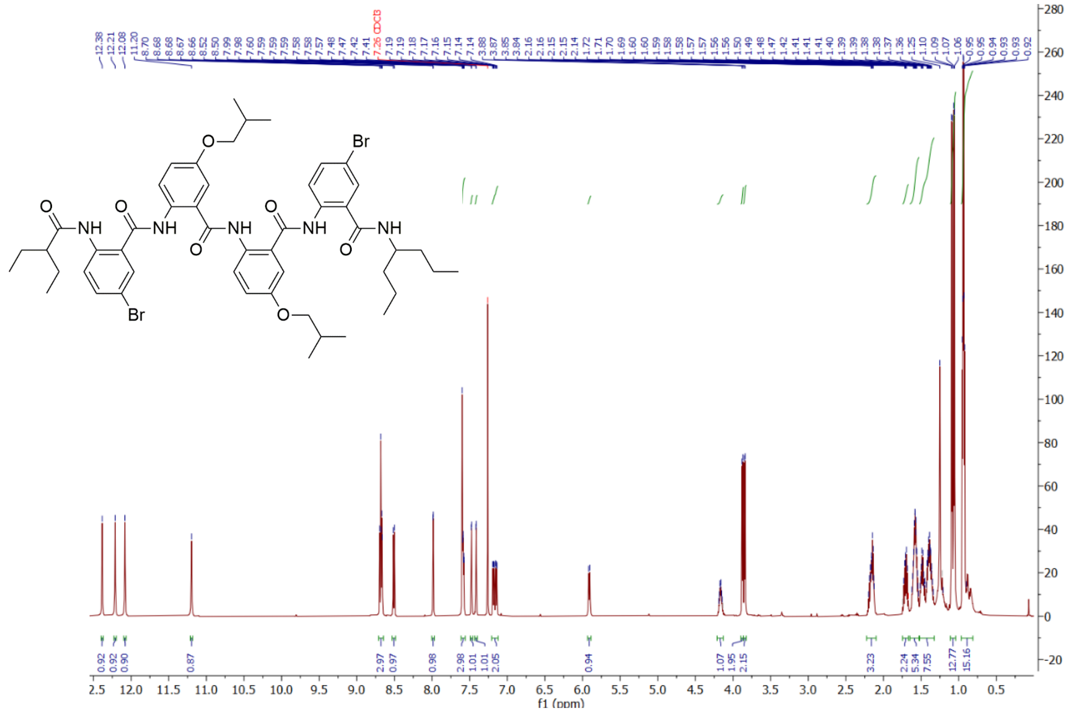

a

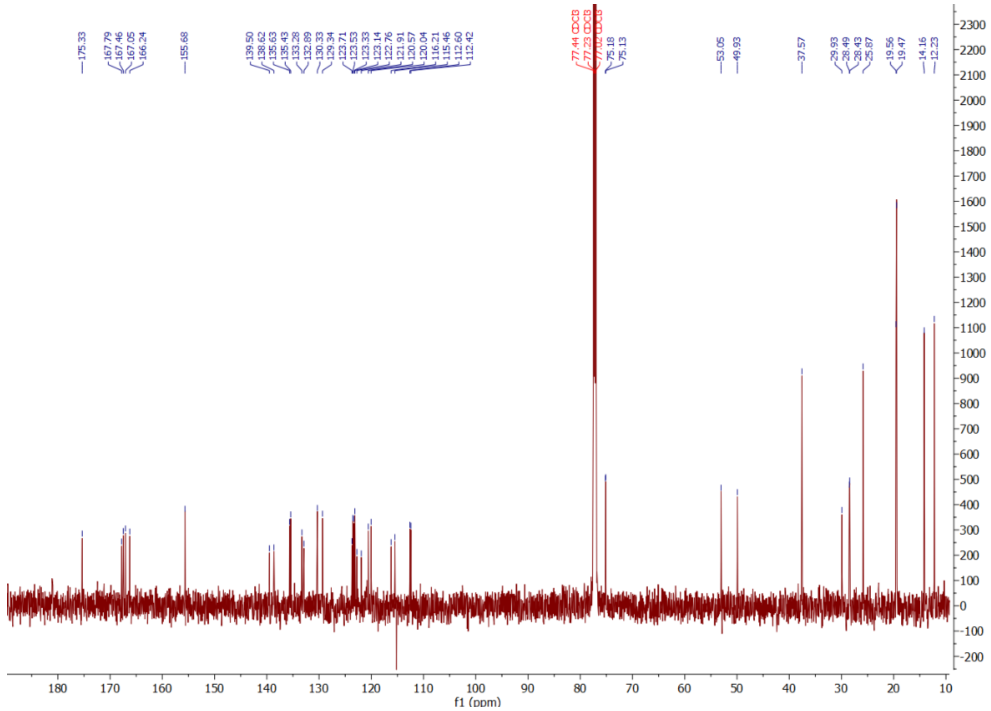

b

Figure S14. (a) ^{1}H NMR of **Baa-Box-Box-Baa** (600 MHz, $CDCl_3$); (b) ^{13}C NMR of **Baa-Box-Box-Baa** (151 MHz, $CDCl_3$).
